# Discovery of an all-donor aromatic [2]catenane†

**DOI:** 10.1039/d0sc04317f

**Published:** 2020-08-24

**Authors:** Tiberiu-M. Gianga, Edwige Audibert, Anamaria Trandafir, Gabriele Kociok-Köhn, G. Dan Pantoş

**Affiliations:** Department of Chemistry, University of Bath BA2 7AY Bath UK g.d.pantos@bath.ac.uk; Materials and Chemical Characterisation Facility (MC2), University of Bath BA2 7AY Bath UK

## Abstract

We report herein the first all-donor aromatic [2]catenane formed through dynamic combinatorial chemistry, using single component libraries. The building block is a benzo[1,2-*b*:4,5-*b*′]dithiophene derivative, a π-donor molecule, with cysteine appendages that allow for disulfide exchange. The hydrophobic effect plays an essential role in the formation of the all-donor [2]catenane. The design of the building block allows the formation of a quasi-fused pentacyclic core, which enhances the stacking interactions between the cores. The [2]catenane has chiro-optical and fluorescent properties, being also the first known DCC-disulphide-based interlocked molecule to be fluorescent.

## Abstract 2

An all-donor [2]catenane has been synthesised *via* dynamic combinatorial chemistry. It features stacked benzodithiophenes which are quasi-pentacyclic through hydrogen bonding.

## Graphical abstract

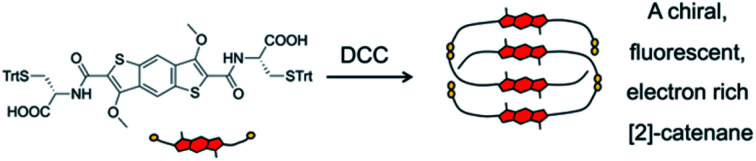

## Introduction

Catenanes have become a curiosity since their advent about half a century ago.^1–3^ Two main strategies have been developed in parallel for their syntheses: metal-templated synthesis^4–9^ and dynamic combinatorial chemistry (DCC).^3,10–16^ After the statistical catenation era (where the interlocked molecules were produced by statistical threading), Jean-Pierre Sauvage pioneered the metal-templated synthesis.^4^ This approach takes advantage of the preorganisation of the ligands around transition metal cations followed by the catenation step (mechanical bond formation). The metal ion can then be removed to obtain a purely organic interlocked molecule.^4,6^ In contrast to this, the active metal templation method, which includes click-chemistry^17^ and other copper catalysed reactions,^6,18,19^ is based on the catalytic properties of some transition metals that have allowed the synthesis of catenanes. Another, less common, strategy is using alkali metal cations as templates.^20^ The metals can also be part of the macrocycle forming the catenane.^21,22^ The strategies described above rely on a template (the metal cation) to bring together the species involved in the catenation process. In contrast to these, there are methods that take advantage of hydrogen and halogen bonding, π–π interactions, and the hydrophobic effect in order to provide the necessary preorganisation in catenane synthesis *via* ring closing metathesis,^23^ amide bond formation or DCC. The Vögtle^24^ and Hunter^25^ groups were able to synthesise catenanes using hydrogen-bonding. Chloride,^26^ bromide^27,28^ and iodide^28^ anions allow halogen bonding (anion templation) that has also led to catenated species. Anion templation was also done *via* hydrogen bonding.^29,30^

DCC exploits the reversible nature of certain covalent bonds (disulphide,^31–33^ imine,^12,34,35^*etc.*) and has emerged as an efficient method for catenane synthesis. In DCC, simple building blocks are mixed together and react until the thermodynamic equilibrium is reached. The catenane formation has been driven by aromatic π–π stacking interactions and the hydrophobic effect.^32,36,37^ The hydrophobic effect is a non-covalent interaction that uses the ability of molecules with hydrophobic cores to “shield” themselves from aqueous media through stacking. The hydrophobic effect has been used in DCC and non-DCC syntheses. Highlights of the latter include examples from the Stoddart group, which have assembled a catenane using the cavity of a beta-cyclodextrin,^38^ and Fujita's group “magical rings” which yielded a [2]catenane quantitatively in the presence of high salt concentration.^21^

**Scheme 1:**
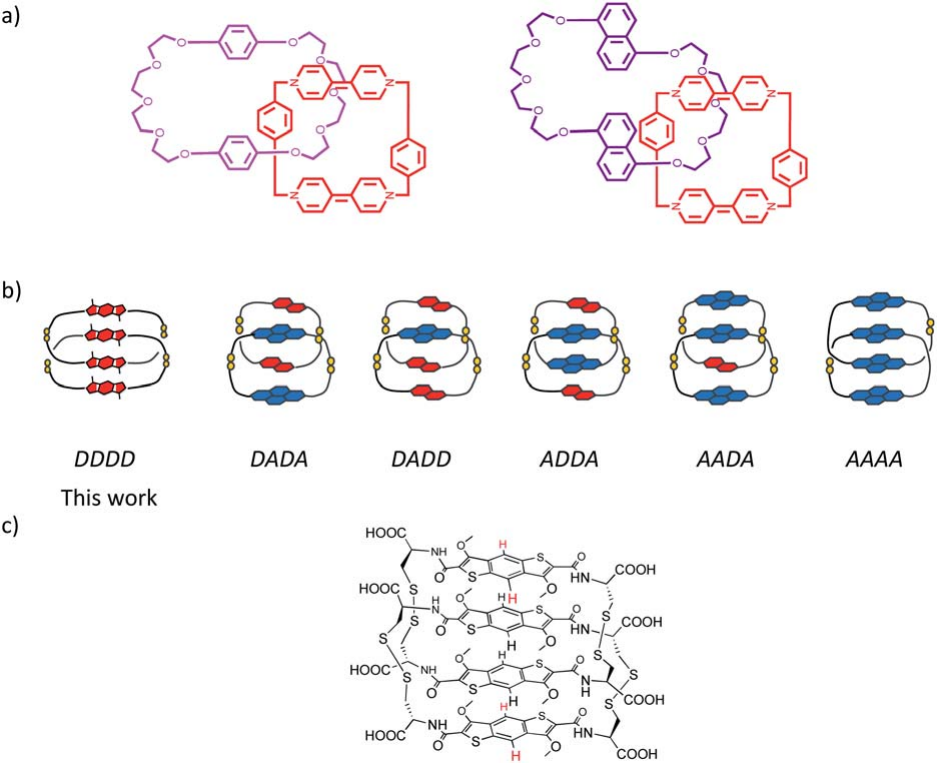
(a) The previously published all-donor [2]catenanes by Stoddart group; (b) the arrangement described in this paper along with the other types of [2]catenanes stacking published by the Sanders group (red – π-donor, dialkoxynaphthalene, blue – π-acceptor, naphthalenediimide) and (c) the chemical structure of the all-donor aromatic [2]catenane synthesised in the paper.

One of the most common types of catenane is the one in which four aromatic units are held together by non-covalent aromatic π–π stacking interactions.^12,21,27,33,37,39–51^ Among these, the studies reported in the literature mostly focus on donor–acceptor-based catenanes in which electron donor and electron acceptor aromatics stack to form a DADA (D = donor, A = acceptor) sequence.^47,52–56^ Of all the possible combinations of four aromatic units stacking in a [2]catenane structure, the all-donor structure has only been reported by Stoddart *et al.* albeit with non-aromatic cores (Scheme 1a).^10^ In that report the all-donor catenane was synthesised *via* a 4e^−^ reduction of a classical DADA [2]catenane. The all-donor catenane is peculiar because of highly unfavourable electronic repulsions of aromatic donor units. Herein, we report the spontaneous self-assembly of an all-donor aromatic [2]catenane from a dynamic combinatorial library (Scheme 1c).

## Results and discussion

**Scheme 2:**
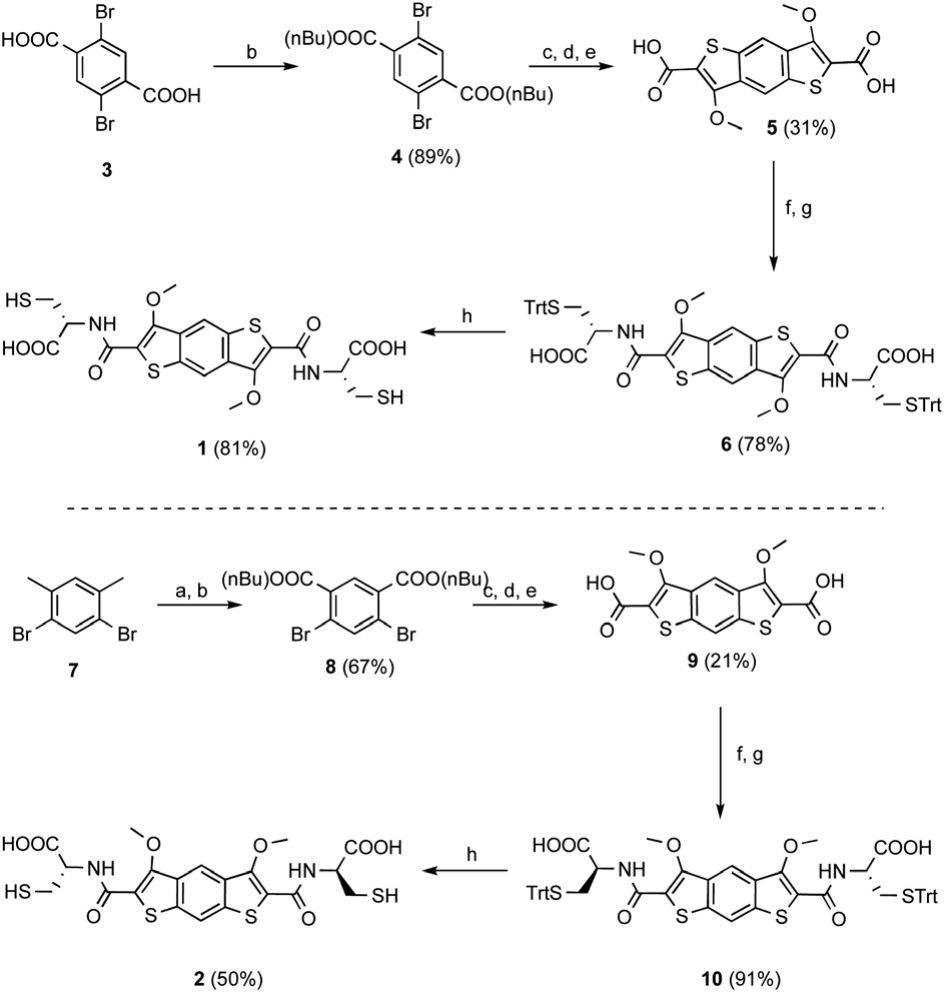
Synthesis of **1** (top) and **2** (bottom) starting from 1,4-dibromoterephthalic acid and 1,5-dibromo-2,4-dimethylbenzene, respectively. The yields under products are over the steps reported in each case. Experimental conditions: (a) KMnO_4_ (2.2 equiv.), ^*t*^BuOH/H_2_O (1 : 1), 120 °C, 12 h; (b) *n*-BuOH, H_2_SO_4_, 120 °C, 12 h; (c) (i) Pd_2_dba_3_ (0.05 equiv.), dppf (0.1 equiv.), 2-ethylhexyl thioglycolate (2.4 equiv.), ^i^Pr_2_EtN (5 equiv.), 100 °C, 15 h; (ii) ^*t*^BuOK (2.9 equiv.), DMF, 100 °C, 3 h; (d) MeI (4 equiv.), K_2_CO_3_ (5 equiv.), DMF, N_2_, r.t., o/n; (e) NaOH 2 M, MeOH, reflux, o/n; (f) EDC HCl (4 equiv.), NHS (4 equiv.), DMF, r.t., o/n; (g) *S*-trityl-l-cysteine (2.2 equiv.), N_2_, r.t., o/n; (h) TFA, Et_3_SiH, r.t., 30 min.

We have designed two isomeric π-donor building blocks for DCC: benzo[1,2-*b*:4,5-*b*′]dithiophene (BDT), **1**, and benzo[1,2-*b*:5,4-*b*′]dithiophene (iBDT), **2**, Scheme 2. The difference between the two building blocks is the connectivity of the benzodithiophene which changes the symmetry of the core: *C*_2h_ for BDT and *C*_2v_ for iBDT.

Computational studies on the two cores indicated that iBDT is slightly less electron rich that BDT, *Q*_*zz*_ = −22 B *vs.* −23 B, respectively. The syntheses of cysteine appended building blocks are described in Scheme 2. They start with either 1,4-dibromoterephthalic acid (for BDT) or 1,5-dibromo-2,4-dimethylbenzene (for iBDT) which are converted to the corresponding esters of BDT and iBDT over three and four steps, respectively. The esters are then hydrolysed to give the dicarboxylic acids **5** and **9** (Scheme 2). The building blocks for dynamic combinatorial libraries (DCLs) are generated by the NHS (*N*-hydroxysuccinimide) activation of the acids followed by coupling with l- or d-*S*(Trt)-Cys-OH and finally trifluoroacetic acid deprotection to give the free thiols **1** and **2** (l-enantiomers, Scheme 2; 1* the d-enantiomer of **1**, Scheme S1†).

**Fig. 1:**
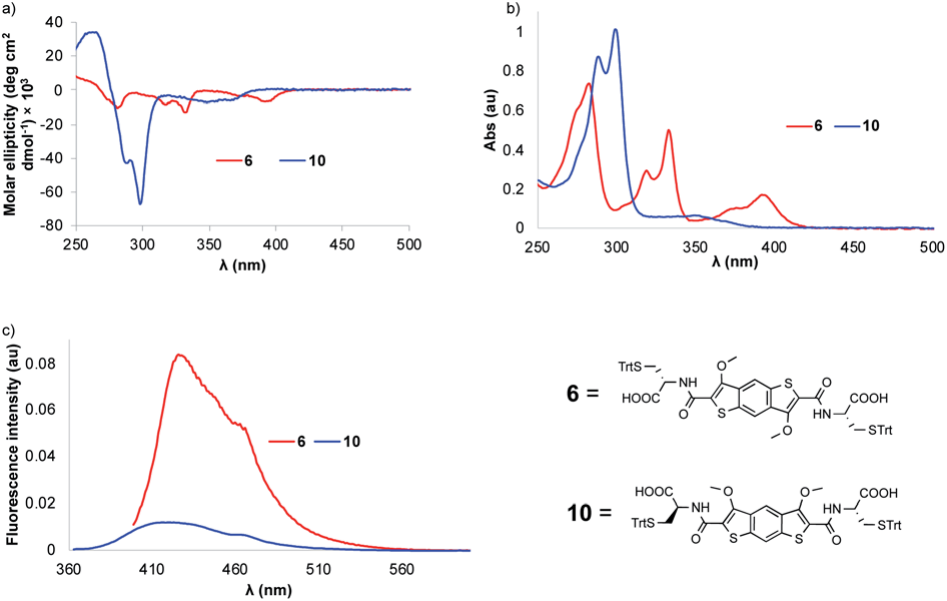
(a) The circular dichroism (CD), (b) the UV-vis and (c) the fluorescence spectra of precursors **6** and **10**.

The absorption profile of the BDT precursor **6** shows a bathochromic shift compared to that of iBDT **10** (*λ*_max_ = 393 nm *vs.* 350 nm, respectively), suggesting a larger band gap for iBDT compared to BDT and a better electron delocalisation in BDT (Fig. 1b). Compound **6** displays a more complex spectrum than **10**, with bands at 333 nm and 282 nm, indicating different electronic properties of the structural isomers. At lower energy, the BDT has a larger extinction coefficient than iBDT (*ε*_393 nm_ = 11 400 L mol^−1^ cm^−1^, *ε*_350 nm_ = 4640 L mol^−1^ cm^−1^, Fig. S28 and S29†). The BDT derivatives are more emissive than their iBDT counterparts – the quantum yield of BDT **6** in CHCl_3_ is 0.075, whereas that of iBDT **10** is 0.008 (Fig. 1c). The chirality of the amino acid is transferred onto both aromatic cores as indicated by their circular dichroism (CD) spectra in CHCl_3_. The molar ellipticities of BDT **6** and iBDT **10** are comparable above 300 nm, however below 300 nm, the BDT's ellipticity is about 7× lower than that of iBDT (Fig. 1a).

**Fig. 2:**
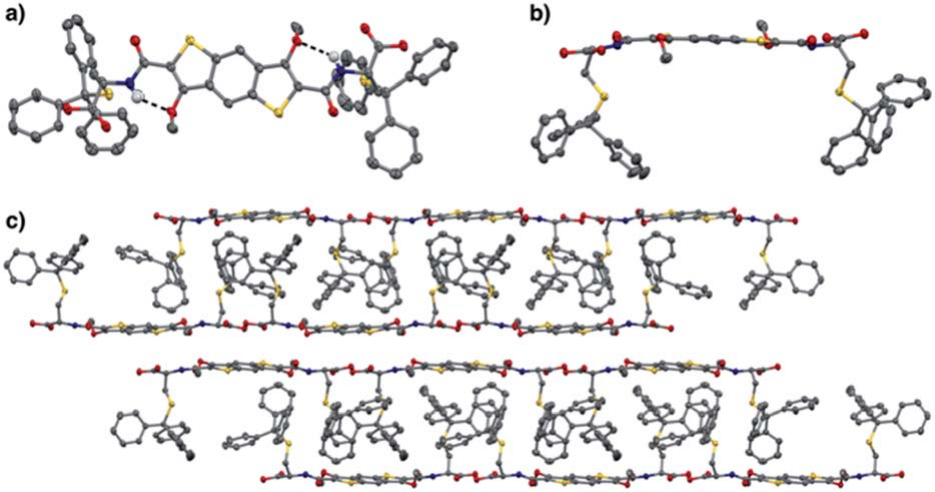
(a) Top view of the X-ray structure of **6** showing the presence of the quasi-pentacyclic core through the hydrogen bonding (dashed line) between NH and OMe. (b) Side view and (c) solid state packing structure of **6** viewed along the crystal *a*-axis. All H atoms, except NH, have been removed for clarity. (C – grey, H – white, O – red, N – blue, S – yellow) – CCDC: 2006236.†

Structural proof for BDT precursor **6** came from solving the X-ray diffraction pattern of crystals grown from a solution of acetone and trifluoroethanol. In the X-ray crystal structure (Fig. 2) a hydrogen bond between the amide NH and methoxy O (N⋯O 2.771(4) Å, ∡N–H–O 138(4)°) is observed. This enables the system to adopt a pseudopentacyclic structure (three aromatic cores from benzodithiophene and two cycles held together by hydrogen bonding between amide and one of the methoxy groups), which makes the amide coplanar with the aromatic core, thus providing a larger π surface.

The solid state supramolecular packing is dominated by the trityl interdigitation and an off-centred aromatic stacking between the BDT cores. Carboxylic acid dimerization is observed between two neighbouring molecules, organising the BDTs in two anti-parallel π-tapes separated by 3.2 Å on average.

**Fig. 3:**
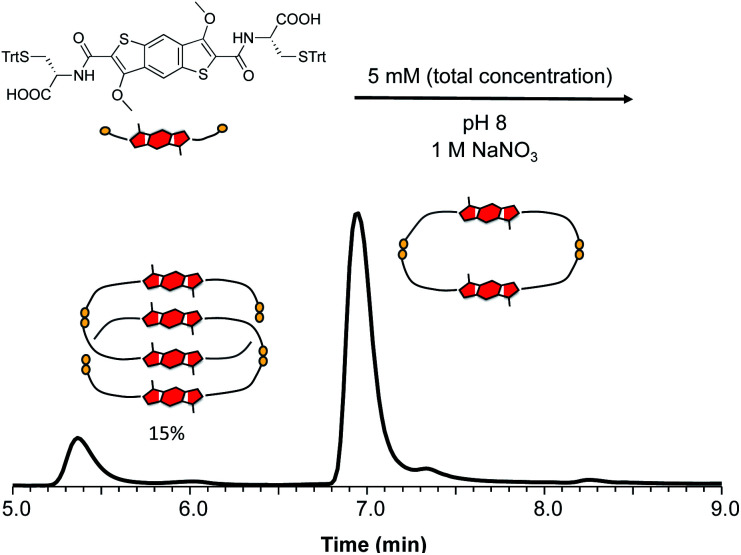
Reverse-phase HPLC analysis of **1** (5 mM concentration) in the presence of 1 M NaNO_3_, showing the formation of an all-donor catenane in 15% yield (based on ^1^H NMR integration).

Individual DCLs of **1** and **2** were prepared by dissolving the building blocks in water at pH 8. The libraries were stirred in closed capped vials, allowing for atmospheric oxidation for at least three days before analysis. The DCL of **1** contained only a homodimer based on HPLC and LC-MS studies. Increasing the ionic strength of the DCL by adding 1 M NaNO_3_ led to the formation of a new species. LC-MS analysis of this DCL (Fig. 3) indicated that the new compound has a shorter retention time despite having double *m*/*z* compared to the homodimer, which suggested an interlocked nature for the species.

The MS and isotope pattern analyses of the peak corresponding to this new compound have indicated that it contains four BDT units, while the MS/MS analysis supports the assumption of an interlocked (catenated) structure of the molecule (*i.e.* no fragment with *m*/*z* higher than a dimer – Fig. S33 and S34† for LC-MS characterisation). The species (a [2]catenane) was isolated for further characterisation.

**Fig. 4:**
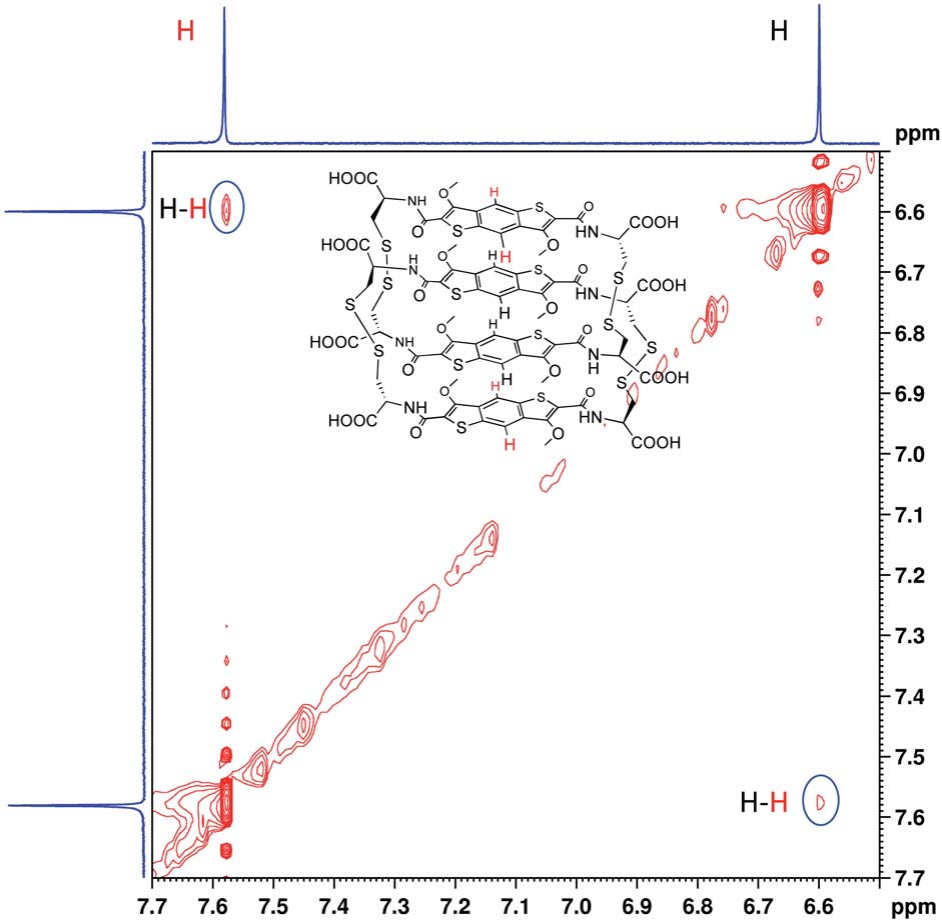
Partial 2D NOESY (red) spectrum (500 MHz, 298 K) of all-donor l-[2]catenane. The solvent (H_2_O) was referenced at 4.79 ppm.

In the ^1^H NMR spectrum we observe that the two signals corresponding to the aromatic cores are more shielded than the analogous ones of the homodimer (Fig. S35 and S36†). This indicates that in the [2]catenane the aromatic protons are shielded due to the close proximity of the π surfaces in structure. The 2D NOESY analysis shows cross peaks between the protons in the aromatic region, suggesting an offset parallel arrangement of the aromatic cores (Fig. 4, S37 and S38†). All the analytical data for this species allows us to designate it as the first ever all-donor catenane obtained through DCC. Attempts to enhance the yield of this [2]catenane by increasing the salt content (NaNO_3_) to 5 and 10 M had a minor influence on its formation, with a maximum yield of 15% being obtained at all salt concentrations (Fig. S39†). The yield was determined by integration of the ^1^H NMR spectrum of a static DCL; the yield determined by integrating the HPLC chromatogram was 12%, the discrepancy is likely due to the broadening of the HPLC peaks. As expected, the enantiomeric library (starting from D-BDT, 1*) gave a quasi-identical distribution with the formation of the enantiomeric all-donor [2]catenane.

**Fig. 5:**
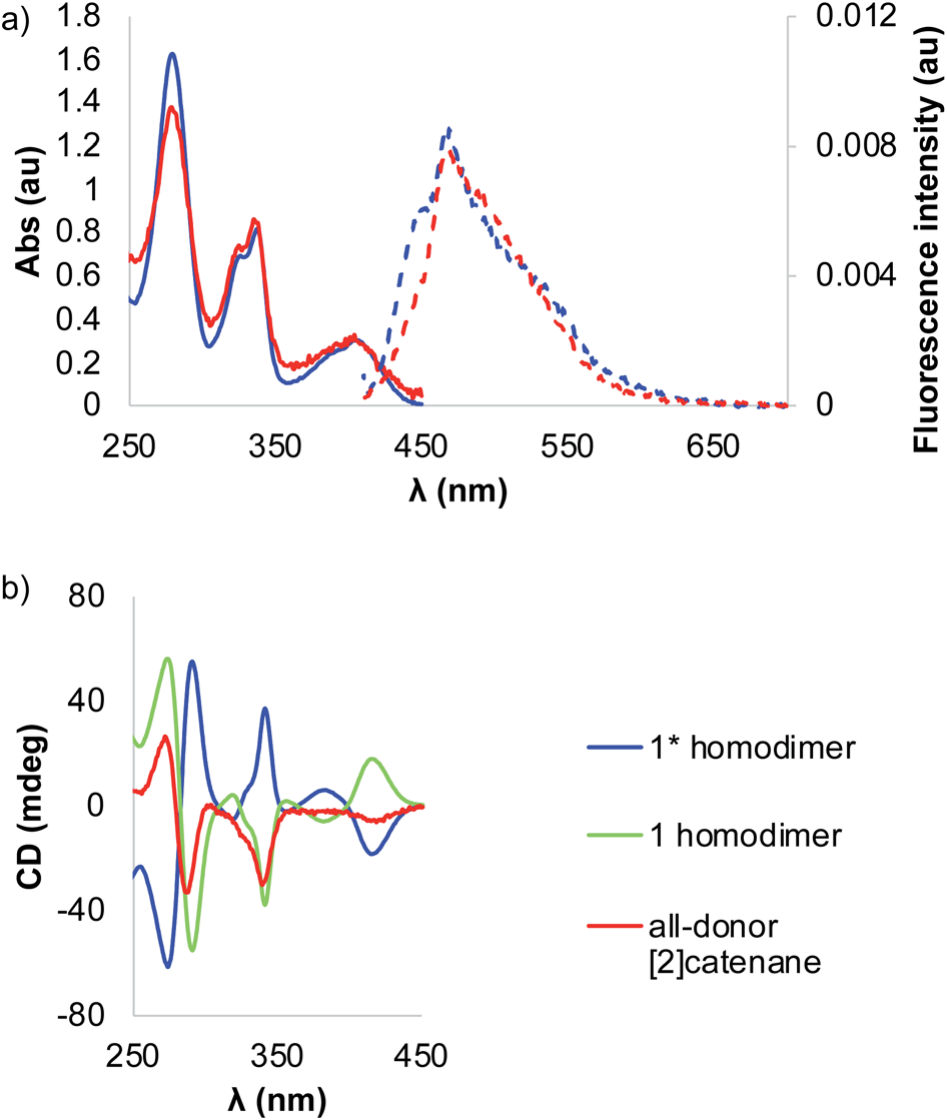
(a) The UV-vis spectra (solid lines) of the 1* homodimer (blue) and all-donor [2]catenane (red) and their respective fluorescence spectra (dotted lines) and (b) the CD spectra of 1* homodimer (blue), **1** homodimer (green) and all-donor [2]catenane (red). The UV-vis and CD spectra are normalised.

The [2]catenane and **1** homodimer have similar absorption spectra with both compounds displaying *λ*_max_ at 407 nm (Fig. 5a). The bands in the spectrum of interlocked molecule are broader than those in homodimer spectrum due to a lower symmetry of the former. The CD spectra of **1** and 1* homodimers are mirror images, as expected, as the compounds are enantiomers. On the other hand, the CD spectrum of the catenane has a different signature with lower ellipticity than the dimers (Fig. 5b). We took advantage of the fluorescent properties of the building blocks and recorded the emission spectra of the dimer and catenane, in which we observed Stoke shifts of 51 and 66 nm, respectively. The quantum yields of both catenane and homodimer **1** have been calculated using fluorescein as standard.

The results indicated that the catenane is almost five times (*Φ*_cat_ = 0.033, *Φ*_dimer_ = 0.007) more fluorescent than the dimer (quantum yields). To the best of our knowledge, this is the first example of an interlocked molecule made *via* disulphide DCC that fluoresces.

A 1 : 1 mixed library containing **1** and 1*, in the absence of the salt, formed at equilibrium three species: **1** homodimer, 1* homodimer and the heterodimer (Fig. S46†). The equilibrated library in which the hydrophobic effect was augmented by adding 1 M NaNO_3_, contains the heterodimer, the enantiomeric homodimers, a heterotetramer, and a hetero all-donor [2]catenane (Fig. S47†). The hetero all-donor mixed chirality [2]catenane was formed in slightly higher proportion than the homochiral [2]catenanes from enantiopure libraries (18% *vs.* 12%, respectively).

**Fig. 6:**
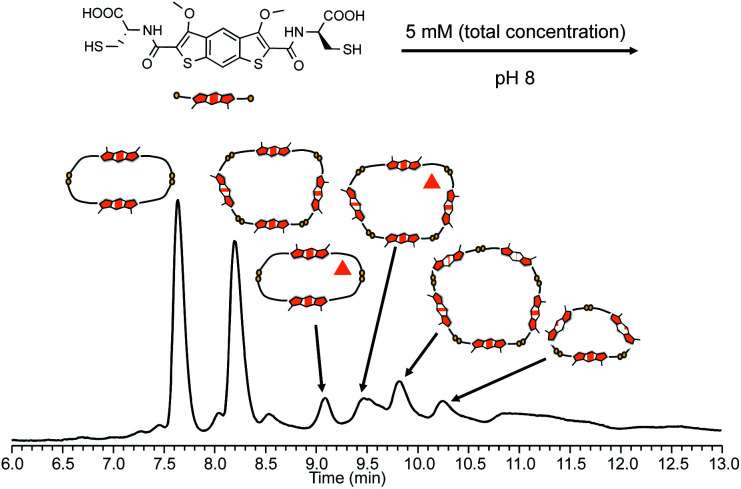
Reverse-phase HPLC analysis of **2** (5 mM concentration) without any additives (the compound with the triangle contains three extra methyl groups).

Analysis of DCLs prepared by dissolving building block **2** (l-iBDT) in water at basic pH (8), followed by oxidation in atmospheric conditions revealed a very different speciation when compared to the analogous DCLs of **1** (Fig. 6). At equilibrium, the library contained two main species: a dimer and a tetramer. The minor species in the DCL consisted of a pentamer, a trimer and two methylated impurities of the dimer and the tetramer, respectively. The tetramer (as well as the pentamer) is a macrocycle and not an interlocked molecule as indicated by the MS/MS analysis where fragments with masses higher than that of a dimer were present (Fig. S54 and S57†).

Addition of 1 M NaNO_3_ changes the library distribution significantly (Fig. S52†). The HPLC analysis of this DCL indicates the formation of oligomeric species as no major peaks have been detected and a gradual increase of the baseline has been observed with increasing MeOH content in the mobile phase.

In order to understand the stark difference in behaviour for DCLs of **1** and **2** we turned our attention to molecular modelling.

**Fig. 7:**
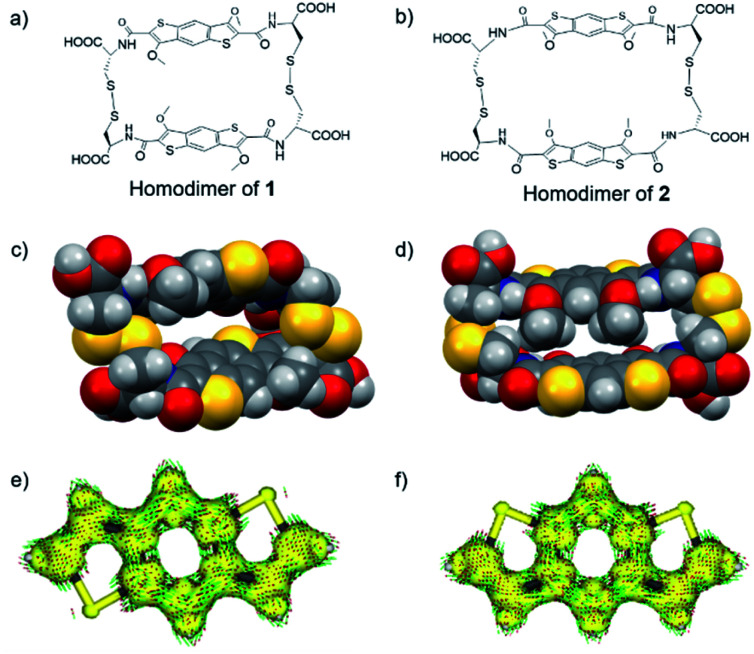
Chemical structures (a and b) and overlapping spheres optimized geometries of homodimers of **1** and **2** (c and d). Anisotropy induced current densities (ACID) plots (isovalue 0.05 e bohr^−3^) of the BDT and iBDT cores (e and f).

The self-complementary stacking interactions between the cores of the isomer building blocks are influenced by the electronic properties of their π systems. The electron delocalisation in the two monomer cores has been analysed by ACID (anisotropy of the current-induced-density) calculations (Fig. 7e and f), which show the flux of delocalised electrons in π-rich molecules. Both cores result to be conjugated and aromatic as shown by a diatropic (clockwise) ring current. Therefore, the electron delocalisation in the BDT and iBDT cores does not yield itself as a decisive reason for the different behaviour of DCL of **1** and **2**.

Furthermore, the geometry optimisation (M06-2X/6-311G/H_2_O) of the stacked homodimeric aromatic cores (without any linkage between them and omitting the OMe substituents) reveals that both core pairs stack in a similar fashion with an average distance between the two cores of 3.256 Å (iBDT) and 3.311 Å (BDT) (Fig. S59†), leading to the conclusion that the interaction between them (at this level of theory) is comparable. The relaxed geometries of the homodimers of **1** and **2** (M06-2X/6-31G/H_2_O) shown in Fig. 7c and d demonstrate the different conformation adopted by the two systems: the BDT structure possesses a cavity in which another aromatic core can be threaded through while the iBDT dimer adopts a self-complementary geometry which closes the gap between the π surfaces. In the case of the latter, we postulate that the presence of the methoxy groups on the same side of the fused tricyclic system imposes steric demands that prevent aromatic stacking in aqueous media. Calculations at the same theory level of the enthalpy of formation for each dimer from its constituent building blocks indicate that the energy release in the formation of the dimer of **1** is 28.6 kcal mol^−1^ larger than the corresponding value for the homodimer of **2**. The difference in the enthalpies of formation and in the geometries adopted by the two dimers could be the reason why the all-donor [2]catenane forms in the DCL of **1** and is not present in the DCL of **2**.

The different outcomes of the DCLs of these isomers show the powerful nature of DCC, how selective it can be, and how narrow the barrier between interlocked and non-interlocked molecules is.

## Conclusions

In conclusion, we reported herein the synthesis and characterisation of the first aromatic all-donor [2]catenane based on a benzodithiophene core using the DCC approach. The key structural feature is the formation of an extended aromatic core, quasi-pentacyclic, through a set of strong NH⋯OMe hydrogen bonds. The catenation of two BDT dimers happens only when the hydrophobic effect is increased by the addition of an inorganic salt. The isomeric iBDT, despite having the same quasi-pentacyclic fused core does not form a [2]catenane due to the relative position of the two sulphur atoms which change the stacking geometry of the cores. The all donor [2]catenane is the first chiral emissive interlocked molecule synthesised *via* DCC that can be used in chiroptical applications, which we endeavour to investigate.

## Conflicts of interest

There are no conflicts to declare.

## Supplementary Material

SC-011-D0SC04317F-s001

SC-011-D0SC04317F-s002
