# Supplementary material for: Discovery of an all-donor aromatic [2]catenane
Source: Chem Sci. 2020 Aug 24;11(35):9685–90. doi: 10.1039/d0sc04317f (PMC8162110; doi:10.1039/d0sc04317f)
Supplement: SC-011-D0SC04317F-s001 [file SC-011-D0SC04317F-s001.pdf]

Electronic Supplementary Material (ESI) for Chemical Science.
This journal is © The Royal Society of Chemistry 2020

Supplementary Materials for

Discovery of an all-donor aromatic [2]catenane

Tiberiu-M. Gianga,[a,b] Edwige Audibert,[a] Anamaria Trandafir,[a] Gabriele Kociok-Köhn,[c] G. Dan Pantoș[a*]

a. Department of Chemistry, University of Bath, BA2 7AY, Bath, U.K.
b. Current address: Beamline B23, Diamond Light Source, Ltd., Chilton, Didcot OX11 0DE, U.K.
c. Materials and Chemical Characterisation Facility (MC2), University of Bath, BA2 7AY, Bath, U.K.

Correspondence to: G.D.Pantos@bath.ac.uk

This PDF file includes:

Materials and Methods
Supplementary Text
Scheme S1
Figs. S1 to S59
Table S1-2

Materials and Methods

All reagents were purchased from commercial suppliers: Acros Organics, Alfa Aesar, Sigma Aldrich, TCI Europe, Fluorochem and used without further purification.

^{1}H and ^{13}C NMR spectra were recorded on 500 MHz Agilent Propulse or 500 MHz Bruker Avance II+ (^{1}H 500 MHz, ^{13}C 125 MHz) instruments. Chemical shifts (δ) are reported in parts per million (ppm). Coupling constants are reported in Hertz (Hz), and signal multiplicity is denoted as singlet (s), doublet (d), doublet of doublet (dd), triplet (t), dt (doublet of triplets), td (triplet of doublets), dtd (doublet of triplets of doublets), h (heptet), multiplet (m) and broad (br). All spectra were acquired at 25 °C and were referenced to the residual solvent peaks. The common solvent impurities in ^{1}H and ^{13}C NMR in small amounts were water, acetone or DMF. COSY, NOESY spectra were recorded on a Bruker AV III 500 MHz fitted with Prodigy (nitrogen-cooled) cryoprobe.

LC-MS studies were carried out on a Thermo Surveyor PDA Plus LC and LCQ classic ESI MS. Data was processed using the XCalibur software. The individual HPLC / LC-MS methods are detailed in the LC-MS Analysis Section. The LC CD data was acquired on a Jasco CD 2095 connected to a SS420x A/D converter.

The HRMS data was acquired on a Bruker MaXis HD ESI-QTOF mass spectrometer for high mass accuracy, coupled to a Thermo Scientific Dionex Ultra High Performance Liquid Chromatography (UPLC) unit.

All the CD data was acquired on an Applied Photophysics Chirascan spectrophotometer equipped with a Peltier temperature controller.

Synthesis of building blocks

Compound **4** was synthesized following the published procedures.[1]

The following compound, **12**, was synthesised using a modified procedure:

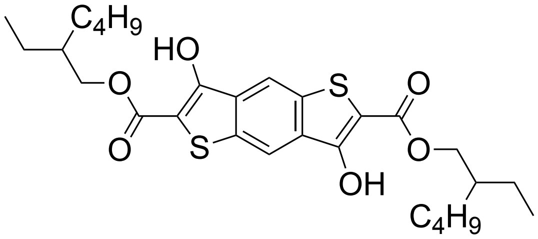

A round-bottom flask was charged with **4** (1.50 g, 3.46 mmoles, 1 equiv), $Pd_2(dba)_3$ (156.2 mg, 0.17 mmoles, 0.05 equiv) and dppf (191.1 mg, 1.34 mmoles, 0.1 equiv) and the mixture was dissolved in DMF (30 mL) while under N_2 atmosphere. $^{i}Pr_2NEt$ (2.97 mL, 17.05 mmoles, 4.97 equiv) and 2-ethylhexyl thioglycolate (1.72 mL, 8.18 mmoles, 2.37 equiv) were added and the reaction mixture was heated at 100 °C for 15 h. The reaction was then allowed to cool to r.t. and $^{t}BuOK$ (1.11 g, 9.90 mmoles, 2.87 equiv) and DMF (8 mL) were added. The reaction was further heated at 100 °C for another 3 h. The system was cooled again to r.t. and the reaction mixture was acidified with 1 M HCl_{aq}, followed by extraction with toluene. The organic fraction was filtered over Celite® and the cake was washed with ethyl acetate. The solvents were removed under reduced pressure and the residue was purified by column chromatography (CH_2Cl_2 : Petroleum Ether 60:40 *v*/*v*), giving the product **12** (1.04 g, 1.95 mmoles, 56%). The ^{1}H NMR spectrum is matching the one reported in literature.[1]

Synthesis of **13**:

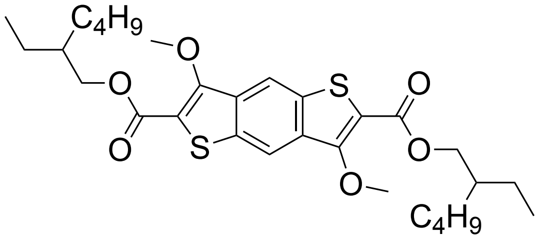

12 (504.1 mg, 0.95 mmoles,1 equiv) was dissolved in DMF (60 mL) under N_2 atmosphere and K_2CO_3 (2.80 g, 20.20 mmoles, 21 equiv) was added. This was stirred half an hour at r.t., then MeI (0.5 mL, 8.00 mmoles, 8.5 equiv) was added and the reaction mixture was further stirred overnight. Once the reaction completed, water was added, and the mixture was extracted with CH_2Cl_2. The organic fraction was dried over anhydrous $MgSO_4$ and the solvent removed under reduced pressure to yield an viscos liquid, which was further dried. The compound was impure at this stage but used as it was in the next steps; the yield could not be determined. ^{1}H NMR (500 MHz, $CDCl_3$): δ 8.26 (s, 2H), 4.27 (dd, J = 5.7, 3.6 Hz, 4H), 4.20 (s, 6H), 1.73 (h, J = 5.9 Hz, 4H). The rest of the protons cannot be assigned because of the complexity due to impurities. ^{13}C NMR (125 MHz, $CDCl_3$): δ 169.6, 162.5, 161.6, 155.5, 140.0, 134.5, 134.3, 122.7, 117.1(3), 117.0(8), 117.0(2), 68.0, 67.8, 62.8, 38.9, 38.9, 36.4, 31.4, 30.5(1), 30.4(7), 29.0, 28.9, 23.9(3), 23.9(0). There are more peaks in the ^{13}C NMR spectrum than expected because of the impurity. TOF MS ASAP+: *m/z* calcd for $C_{30}H_{42}O_6S_2$ $[M+H]^+$ 563.2501, found 563.2504.

Synthesis of **5**:

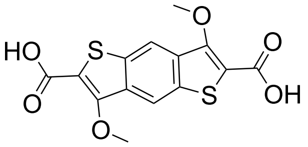

A round-bottom flask was charged with **13** (474.5 mg) dissolved in acetone (2 mL), followed by addition of MeOH (80 mL) and 2 M $NaOH_{aq}$ (40 mL). The reaction mixture was refluxed overnight. Upon cooling, the solution was acidified with concentrated HCl to give a yellow precipitate. The solid was filtered off and washed with CH_2Cl_2 and acetone, then collected and dried to give **5** as a yellow solid (178.5 mg, 0.53 mmoles, 56% over 2 steps). ^{1}H NMR (500 MHz, DMSO-*d$_6$*): δ 13.46 (br, 2H), 8.50 (s, 2H), 4.09 (s, 6H). ^{13}C NMR (125 MHz, DMSO-*d$_6$*): δ 162.9, 154.8, 134.5, 134.0, 118.8, 117.9, 63.0. TOF MS ASAP+: *m/z* calcd for $C_{14}H_{10}O_6S_2$ $[M+H]^+$ 338.9997, found 338.9996.

Synthesis of **14**:

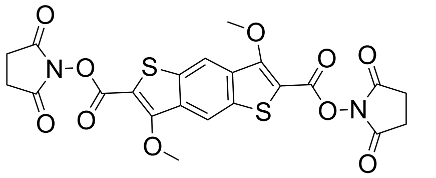

In a round-bottom flask, compound **5** (99.7 mg, 0.30 mmoles, 1 equiv) and *N*-hydroxysuccinimide (133.4 mg, 1.16 mmoles, 4 equiv) were dissolved in dry DMF (10 mL) and cooled to 0 °C using an ice bath. Once cooled, EDC·HCl (226.8 mg, 1.18 mmoles, 4 equiv) was added and the mixture was stirred for 15 minutes in the melting ice bath. The ice bath was removed and the reaction mixture was further stirred overnight at r.t. The solvent was evaporated to dryness and the residue was re-dissolved in a small amount of acetone. The suspension formed was added dropwise to a vigorously stirred 1 M HCl_{aq} solution. The precipitate obtained was filtered off under vacuum and dried to obtain a brownish solid (127.4 mg, 0.24 mmoles, 81%). ^{1}H NMR (500 MHz, DMSO-*d$_6$*): δ 8.86 (s, 2H), 4.21 (s, 6H), 2.89 (s, 8H). ^{13}C NMR (125 MHz, DMSO-*d$_6$*): δ 170.6, 159.4, 157.3, 135.4, 134.1, 119.7, 110.6, 63.7, 26.0. FTMS+pNSI: *m/z* calcd for $C_{22}H_{16}N_2O_{10}S_2$ $[M+NH_4]^+$ 550.0585, found 550.0580.

Synthesis of **6**:

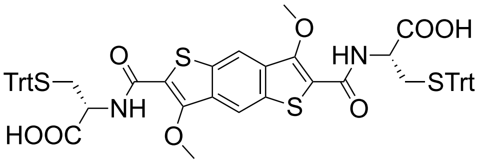

A flame-dried round-bottom flask was charged with **14** (50.5 mg, 0.1 mmoles, 1 equiv) dissolved in dry DMF (10 mL) and *S*-trityl-L-cysteine (76.0 mg, 0.21 mmoles, 2.1 equiv) was added under N_2. Dry Et_3N (0.1 mL) was added and the reaction mixture was stirred overnight at r.t. The solvent was evaporated to dryness and the residue was re-dissolved in a small volume of acetone. The suspension was precipitated dropwise into a vigorously stirred 1 M HCl_{aq} solution. The yellow precipitate was filtered off under vacuum and dried (92.3 mg, 0.09 mmoles, 96%). ^{1}H NMR (500 MHz, DMSO-*d$_6$*): δ 13.19 (br, 2H), 8.70 (s, 2H), 8.42 (d, J = 7.6 Hz, 2H), 7.35-7.15 (m, 30H), 4.55 (td, *J* = 7.3, 4.6 Hz, 2H) 4.16 (s,

6H), 2.79 (dd, *J* = 12.3, 7.0 Hz, 2H). The other beta proton of cysteine moiety is under the satellite of the deuterated solvent. ^{13}C NMR (125 MHz, DMSO-*d*$_6$): δ 176.3, 165.4, 155.1, 149.3, 138.7, 137.2, 134.3, 133.2, 132.1, 71.3, 67.7, 56.5, 35.89. FTMS-pNSI: *m/z* calcd for $C_{58}H_{48}N_2O_8S_4$ $[M-H]^-$ 1027.2221, found 1027.2198.

Synthesis of **1**:

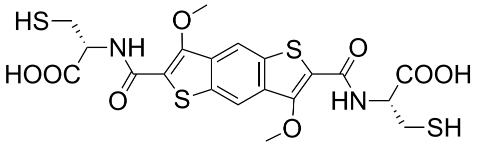

6 (80.0 mg, 0.08 mmoles, 1 equiv) was dissolved in CH_2Cl_2 (3 mL) to which TFA (3 mL) and $SiEt_3H$ (0.3 mL) were added. The reaction mixture was stirred 30 minutes at r.t. and the solvents were removed under pressure. The residue was suspended in Et_2O to give a viscos liquid. The solvent was removed again, and the residue re-dissolved in Et_2O to ensure the precipitation of the desired product. To this, *n*-hexane was added and the solid was filtered off and further washed with *n*-hexane to yield **1** as an yellow solid that was dried (34.1 mg, 0.06 mmoles, 81%). ^1H NMR (500 MHz, DMSO-*d*$_6$): δ 13.29 (br, 2H), 8.69 (s, 2H), 8.47 (d, *J* = 7.4 Hz, 2H), 4.76 (dt, *J* = 7.4, 4.9, 2H) 4.20 (s, 6H). 3.15-3.00 (m, 4H). ^{13}C NMR (125 MHz, DMSO-*d*$_6$): δ 171.6, 160.8, 150.5, 134.0, 132.6, 124.4, 118.2, 63.0, 54.3, 26.2. FTMS-pNSI: *m/z* calcd for $C_{20}H_{19}N_2O_8S_4$ $[M-H]^-$ 543.0030, found 543.0032.

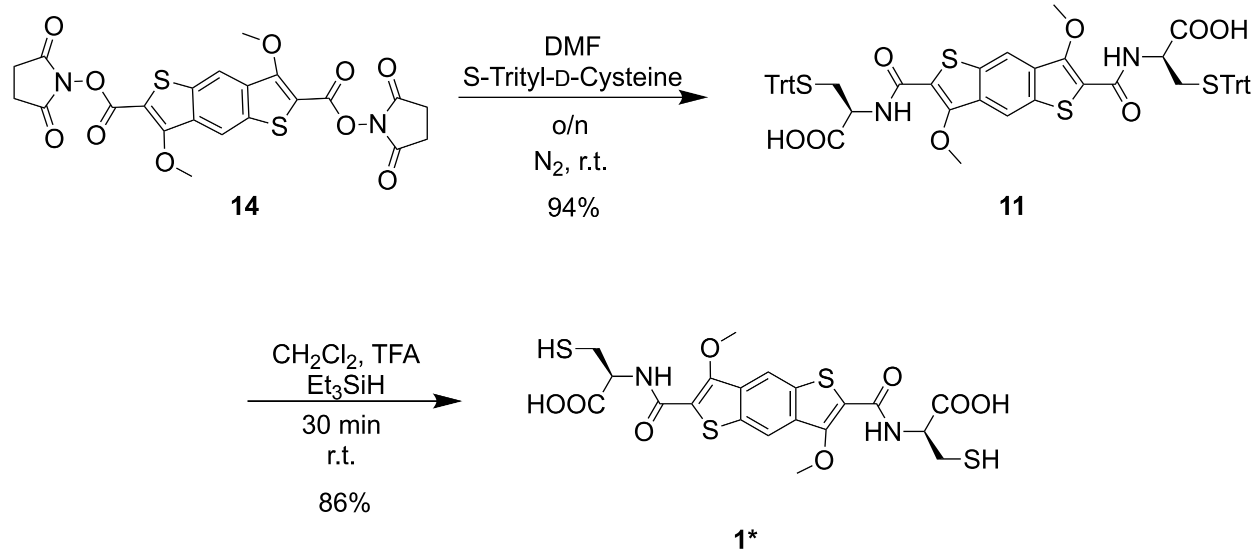

Supplementary Scheme S1. Synthesis of **1***.

Synthesis of **11**:

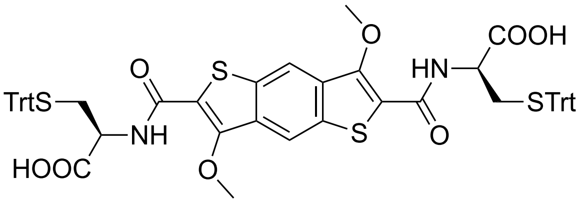

In a flame-dried round-bottom flask, *S*-trityl-D-cysteine (150.5 mg, 0.41 mmoles, 2.2 equiv) was added to a solution of **14** (100.6 mg, 0.19 mmoles, 1 equiv) in DMF (10 mL) under N_2. Et_3N (0.5 mL) was added and the reaction was stirred overnight at r.t. The solvent was removed under pressure and a small volume of acetone was added to the mixture, which was precipitated dropwise into a 1 M HCl solution vigorously stirred. The pale yellow precipitate was collected by filtration and dried (170.1 mg, 0.18 mmoles, 94%). ^{1}H NMR (500 MHz, DMSO-*d*$_6$): δ 13.20 (br, 2H), 8.71 (s, 2H), 8.43 (d, *J* = 7.2 Hz 2H), 7.40 – 7.15 (m, 30H), 4.56 (td, *J* = 7.2, 4.6 Hz, 2H), 4.17 (s, 6H), 2.80 (dd, *J* = 12.2, 7.1 Hz, 2H). The other beta protons are under the satellite of DMSO peak. ^{13}C NMR (125 MHz, DMSO-*d*$_6$): δ 171.6, 160.7, 150.4, 144.6, 134.0, 132.6, 129.5, 128.5, 127.3, 124.0, 118.3, 66.6, 63.0, 51.7, 33.73. FTMS + pNSI: *m/z* calcd for $C_{58}H_{48}N_2O_8S_4$ $[M+Na]^+$ 1051.2186, found 1051.2205.

Synthesis of **1***:

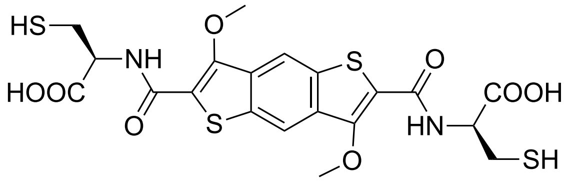

In a round-bottom flask, **11** (149.8 mg, 0.15 mmoles) was dissolved in CH_2Cl_2 (3 mL) followed by addition of TFA (3 mL) and $SiEt_3H$ (0.3 mL). The reaction mixture was stirred 30 minutes at r.t. and the volatiles were subsequently removed. Et_2O was added, removed under pressure and re-dissolved in Et_2O to ensure the precipitation of the desired product. To this, *n*-hexane was added and the solid was filtered and further washed with *n*-hexane to yield **1*** as a pale yellow precipitate that was dried (68.0 mg, 0.12 mmoles, 86%). ^{1}H NMR (500 MHz, DMSO-*d*$_6$): δ 13.30 (br, 2H), 8.70 (s, 2H), 8.48 (d, *J* = 7.4, 2H), 4.77 (dt, *J* = 7.4, 4.9 Hz, 2H), 4.20 (s, 6H), 3.15 – 3.01 (m, 4H). ^{13}C NMR (125 MHz, DMSO-*d*$_6$): δ 171.7, 160.8, 150.5, 134.0, 132.6, 124.4, 118.2, 63.0, 54.3, 26.3. FTMS + pNSI: *m/z* calcd for $C_{20}H_{19}N_2O_8S_4$ $[M+H]^+$ 545.0175, found 545.0168.

Compound **15**, the carboxylic acid corresponding to compound **7**, was synthesized following the published procedures.[2]

Synthesis of **8**:

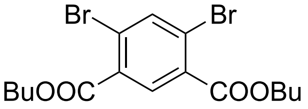

15 (5.57 g, 17.2 mmoles, 1 equiv) was added in a RBF followed by addition of *n*-BuOH (81 mL) and conc. H_2SO_4 (1.2 mL) and refluxed overnight. After cooling the reaction mixture, most of *n*-BuOH was removed in vacuo followed by neutralisation with sat. $NaHCO_3$, then residue was extracted with EtOAc and washed with water then dried over $MgSO_4$. The next step was column purification (Petroleum ether, followed by CH_2Cl_2) and the solvents were evaporated to yielding a white compound (3.75 g, 8.61 mmoles, 50%). ^1H NMR (500 MHz, $CDCl_3$): δ 8.25 (s, 1H), 8.02 (s, 1H), 4.36 (t, *J* = 6.6 Hz, 4H), 1.76 (m, 4H), 1.48 (m, 4H), 0.99 (t, *J* = 7.4 Hz, 6H). ^{13}C NMR (125 MHz, $CDCl_3$): 164.7, 139.7, 133.8, 131.3, 125.5, 66.0, 30.5, 19.2, 13.7. FTMS + p NSI: *m/z* calcd for $C_{16}H_{20}Br_2O_4$ $[M+H]^+$ 434.9801, found 434.9791.

Synthesis of **16**:

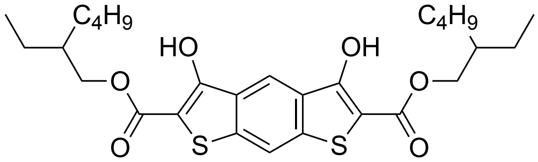

8 (3.75 g, 8.61 mmoles, 1 equiv), $Pd_2(dba)_3$ (0.39 mg, 0.43 mmoles, 0.05 equiv) and dppf (0.48 mg, 0.86 mmoles, 0.1 equiv) were dissolved in DMF (48 mL) under N_2. To this mixture, $^iPr_2NEt$ (7.4 mL, 42.19 mmoles, 4.9 equiv) and 2-ethylhexyl thioglycolate (4.60 mL, 21.52 mmoles, 2.50 equiv) were added and the resulting solution was heated at 100 °C for 15 h. After cooling to r.t., ^tBuOK (2.80 g, 24.97 mmoles, 2.90 equiv) and DMF (20.2 mL) were added and the solution reheated to 100 °C for further 3 h. After cooling to r.t., 1 M HCl was added to acidify the solution, followed by extraction with toluene, filtration over Celite and washings with ethyl acetate. After removing the solvent, the compound was purified by column chromatography (CH_2Cl_2 : Petroleum Ether 20:80 *v*/*v*, then CH_2Cl_2 : Petroleum Ether 80:20 *v*/*v*), giving compound **16** (3.29 g, 6.16 mmoles, 72%). ^1H NMR (500 MHz, $CDCl_3$): δ 10.25 (s, 2H), 8.55 (s, 1H), 8.06 (s, 1H), 4.30 (dd, *J* = 5.8, 2.9 Hz, 4H), 1.74 (h, *J* = 6.2 Hz, 2H), 1.51 – 1.28 (m, 14H), 1.00 – 0.80 (m, 14H). ^{13}C NMR (125 MHz, $CDCl_3$) δ 167.15, 139.10, 128.56, 117.74, 116.97, 67.87, 38.84, 30.45, 28.93, 23.89, 22.94, 14.02, 11.07. + p CI Full MS: *m/z* calcd for $C_{28}H_{38}O_6S_2$ $[M+NH_4]^+$ 552.2, found 552.2.

Synthesis of **17**:

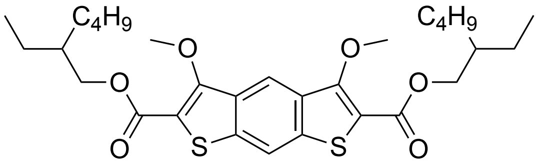

In a round-bottom flask, **16** (3.00 g, 5.60 mmoles,1 equiv) was dissolved in DMF (187 mL) under N_2 atmosphere followed by addition of K_2CO_3 (3.88 g, 28.68 mmoles, 5 equiv). After stirring half an hour at r.t., MeI (1.4 mL, 22.48 mmoles, 4 equiv) was added and further stirred overnight. Water was added, and the compound was extracted with CH_2Cl_2. The organic phase was dried over $MgSO_4$ anhydrous and the solvent removed *in vacuo* to yield an oily liquid, which was dried under high pressure. At this stage the compound was impure but used as it was; no yield was determined at this stage. ^{1}H NMR (500 MHz, $CDCl_3$): δ 8.39 (d, *J* = 0.9 Hz, 1H), 8.08 (d, *J* = 0.9 Hz, 1H), 4.26 (dd, *J* = 5.7, 4.3 Hz, 4H), 4.23 (s, 6H), 1.72 (h, *J* = 6.1 Hz, 4H). The rest of the protons cannot be assigned because of the complexity due to impurities. ^{13}C NMR (125 MHz, $CDCl_3$): δ 169.4(9), 162.5(2), 161.7(2), 156.5(7), 145.6(9), 137.7(8), 132.2(7), 132.0(2), 122.6(0), 117.3(2), 116.8(7), 116.4(4), 114.9(1), 68.5(7), 67.8(3), 67.7(0), 63.0(7), 62.9(3), 61.9(6), 38.9(2), 38.6(0), 36.4(7), 31.4(4), 30.5(2), 30.2(4), 30.1(9), 28.9(7), 28.6(5), 23.9(4), 23.6(7), 22.9(9), 22.8(4), 20.7(2), 14.0(6), 13.8(8), 11.1(0), 10.8(9). There are more peaks in the ^{13}C NMR spectrum than expected because of the impurity. + p APCI corona: *m/z* calcd for $C_{30}H_{42}O_6S_2$ $[M+H]^+$ 563.2496, found 563.2489.

Synthesis of **9**:

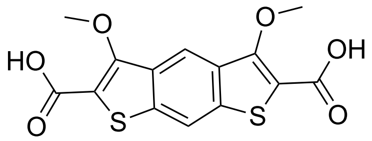

In a round-bottom flask, **17** (6.65 g) was dissolved in acetone (5 mL), followed by addition of MeOH (400 mL) and 2 M NaOH (200 mL); the reaction was stirred overnight at reflux. Upon cooling, concentrated HCl was added until the pH became acidic and a yellow solid precipitated. The precipitate was filtered and washed with CH_2Cl_2 and acetone. The precipitate was collected and dried to yield **9** as a pale yellow solid (0.56 g, 1.66 mmoles, 30% over 2 steps). ^{1}H NMR (500 MHz, DMSO-d_6): δ 13.46 (br, 2H), 8.56 (s, 1H), 8.24 (s, 1H), 4.13 (s, 6H). ^{13}C NMR (125 MHz, DMSO-d_6): δ 162.9, 155.7, 137.0, 132.2, 118.0, 116.7, 116.5, 63.1. FTMS - p NSI: *m/z* calcd for $C_{14}H_{10}O_6S_2$ $[M-H]^+$ 336.9846, found 338.9843.

Synthesis of **18**:

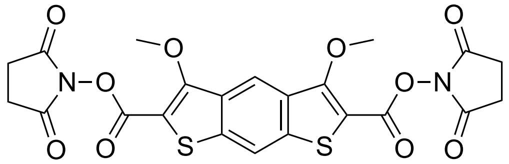

Compound **9** (0.56 g, 1.66 mmoles, 1 equiv) and *N*-hydroxysuccinimide (0.77 g, 6.67 mmoles, 4 equiv) were dissolved in DMF (56 mL) and cooled to 0 °C. EDC·HCl (1.03 g, 6.64 mmoles, 4 equiv) was added and the reaction was stirred for 15 minutes in the melting ice bath. The reaction was further stirred overnight at r.t. The solvent was removed under reduced pressure and a small amount of acetone was added. The suspension formed was added dropwise to a 1 M HCl solution vigorously stirred. The precipitate obtained was collected by vacuum filtration and dried under reduce pressure to obtain a pale yellow precipitate (0.86 g, 1.61 mmoles, 97%). ^{1}H NMR (500 MHz, DMSO-*d*$_6$): δ 8.82 (s, 1H), 8.57 (s, 1H), 4.25 (s, 6H), 2.89 (s, 8H). ^{13}C NMR (125 MHz, DMSO-*d*$_6$): δ 170.7, 160.6, 157.3, 139.2, 131.3, 119.3, 119.0, 107.8, 64.0, 26.0. FTMS + p APCI corona: *m/z* calcd for $C_{22}H_{16}N_2O_{10}S_2$ $[M]^+$ 532.0241, found 532.0241.

Synthesis of **10**:

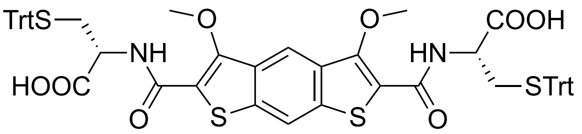

In a flame-dried round-bottom flask, *S*-trityl-L-cysteine (527.8 mg, 1.45 mmoles, 2.2 equiv) was added to a solution of **18** (352.8 mg, 0.66 mmoles, 1 equiv) in DMF (30 mL) under N_2. Et_3N (1.8 mL) was added and the reaction was stirred overnight at r.t. The solvent was removed under pressure and a small volume of acetone was added to the mixture, which was precipitated dropwise into a 1 M HCl solution vigorously stirred. The pale yellow precipitate was collected by filtration and dried (633.3 mg, 0.62 mmoles, 94%). ^{1}H NMR (500 MHz, DMSO-*d*$_6$): δ 13.18 (br, 2H), 8.70 (s, 1H), 8.48 – 8.37 (m, 3H), 7.34 – 7.15 (m, 30H), 4.55 (td, *J* = 7.2, 4.6 Hz, 2H), 4.20 (s, 6H), 2.79 (dd, *J* = 12.2, 7.0 Hz, 2H), 2.61 (dd, *J* = 12.2, 4.5 Hz, 2H). ^{13}C NMR (125 MHz, DMSO-*d*$_6$): δ 171.5, 160.7, 151.1, 144.5, 136.2, 130.9, 129.5, 129.5, 129.4, 128.5, 127.3, 122.4, 66.5, 63.1, 51.7. FTMS – p NSI: *m/z* calcd for $C_{58}H_{48}N_2O_8S_4$ $[M-2H]^{2-}$ 513.1074, found 513.1084.

Synthesis of **2**:

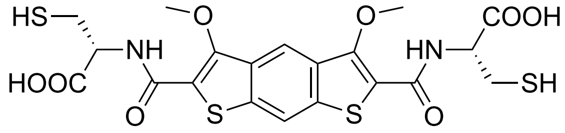

In a round-bottom flask, **10** (124.1 mg, 0.12 mmoles) was dissolved in CH_2Cl_2 (2 mL) followed by addition of TFA (2 mL) and $SiEt_3H$ (0.2 mL). The reaction mixture was stirred 30 minutes at r.t. and the volatiles were subsequently removed. Et_2O was added, removed under pressure and the residue re-dissolved in Et_2O to ensure the precipitation of the desired product. To this, *n*-hexane was added and the solid was filtered and further washed with *n*-hexane to yield **2** as a pale yellow precipitate that was dried (32.5 mg, 0.06 mmoles, 50%). ^{1}H NMR (500 MHz, DMSO-*d*$_6$): δ 8.69 (s, 1H), 8.47 (d, *J* = 7.4

Hz, 2H), 8.43 (s, 1H), 4.77 (dt, *J* = 7.4, 4.9 Hz, 2H), 4.24 (s, 6H), 3.07 (dtd, *J* = 19.2, 8.9, 8.4, 4.9 Hz, 4H). Due to low amount, a ^{13}C NMR was not recorded. – Q-TOF: *m/z* calcd for $C_{20}H_{19}N_2O_8S_4$ $[M-H]^-$ 543.0030, found 543.0025.

NMR spectra

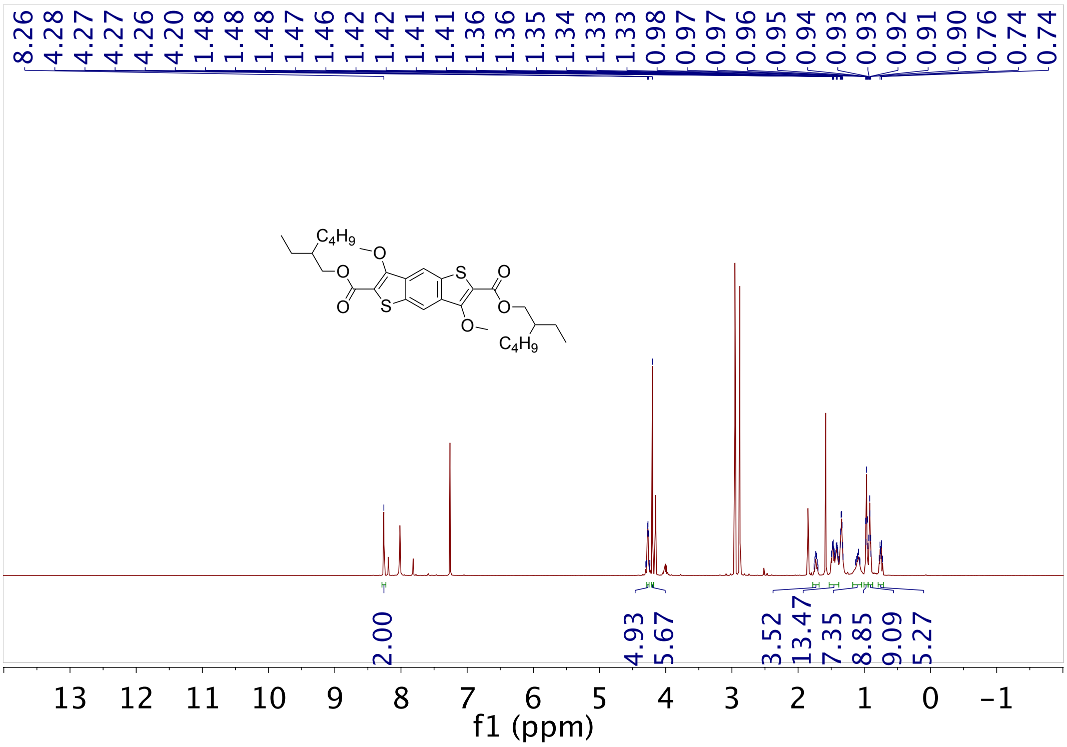

Supplementary Figure S1. ^{1}H NMR spectrum of **13** in $CDCl_3$.

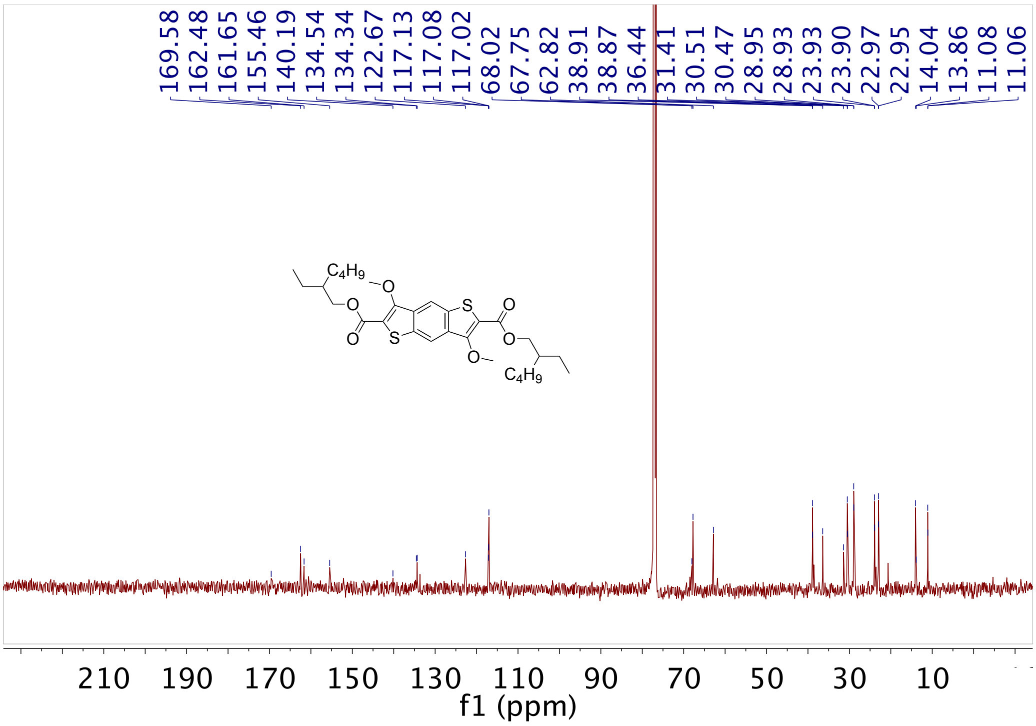

Supplementary Figure S2. ^{13}C NMR spectrum of **13** in $CDCl_3$.

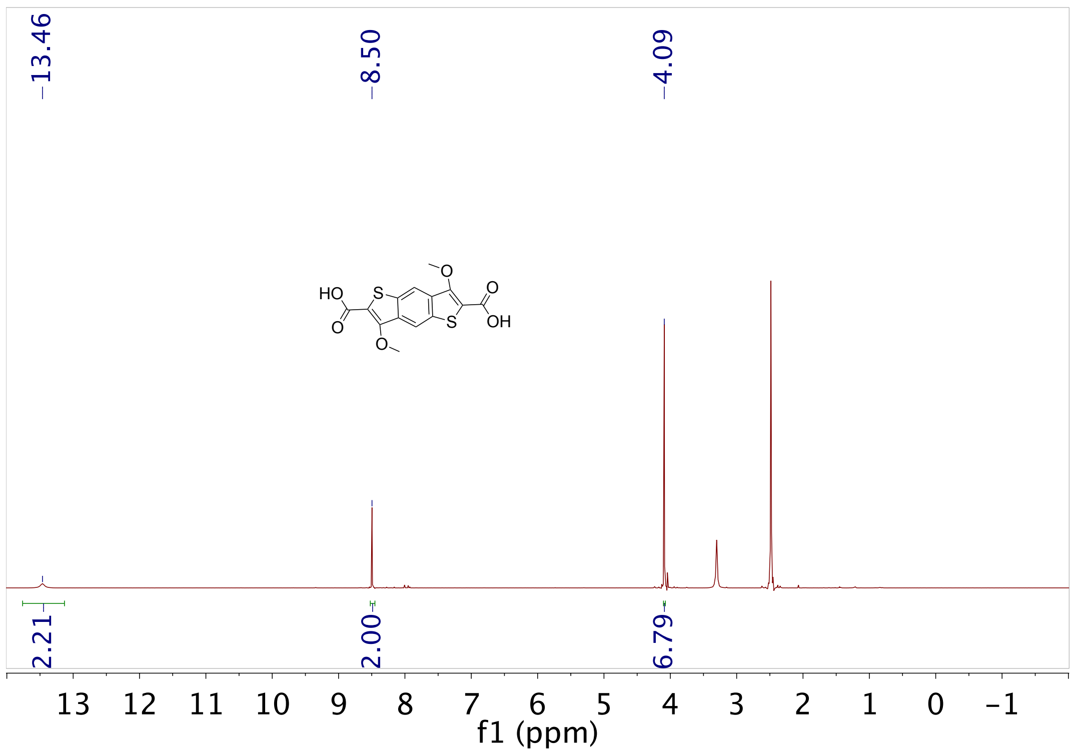

Supplementary Figure S3. ^{1}H NMR spectrum of **5** in DMSO-d_6.

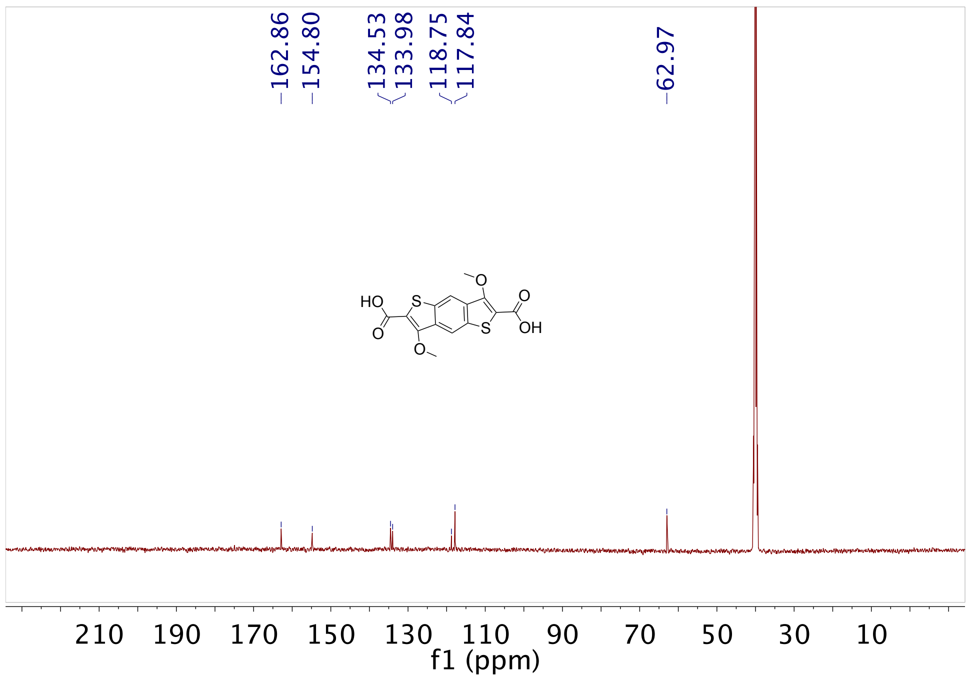

Supplementary Figure S4. ^{13}C NMR spectrum of **5** in DMSO-d_6.

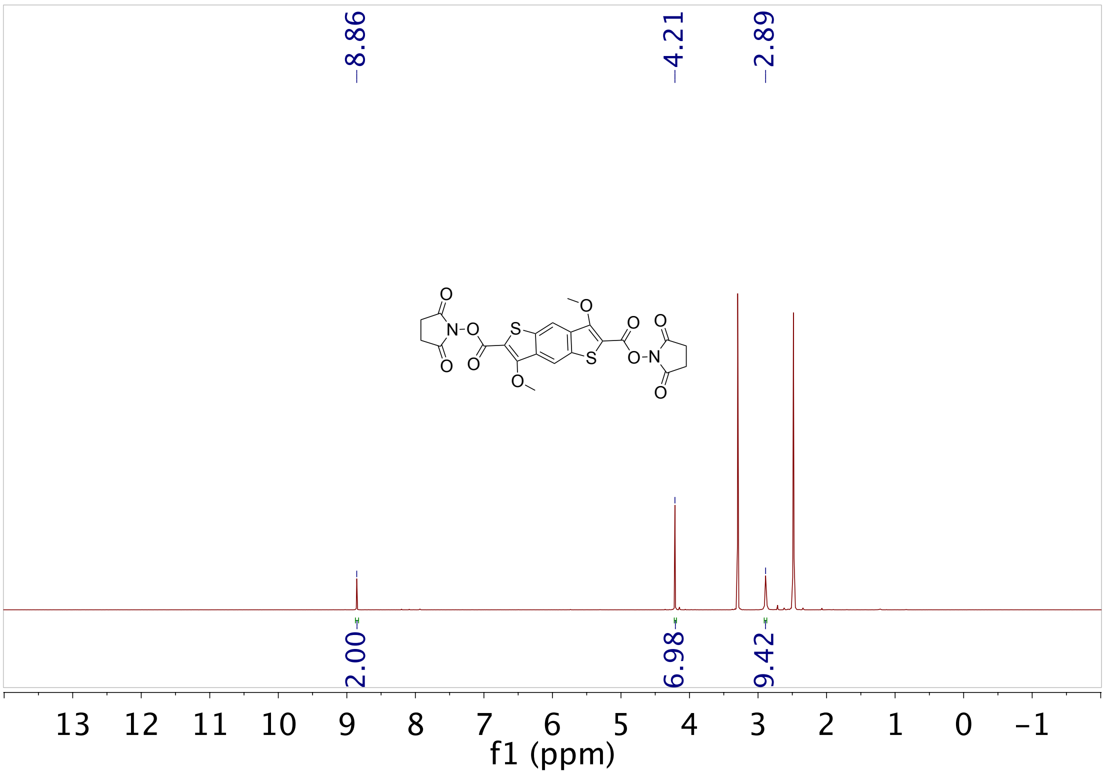

Supplementary Figure S5. ^{1}H NMR spectrum of **14** in DMSO-d_6.

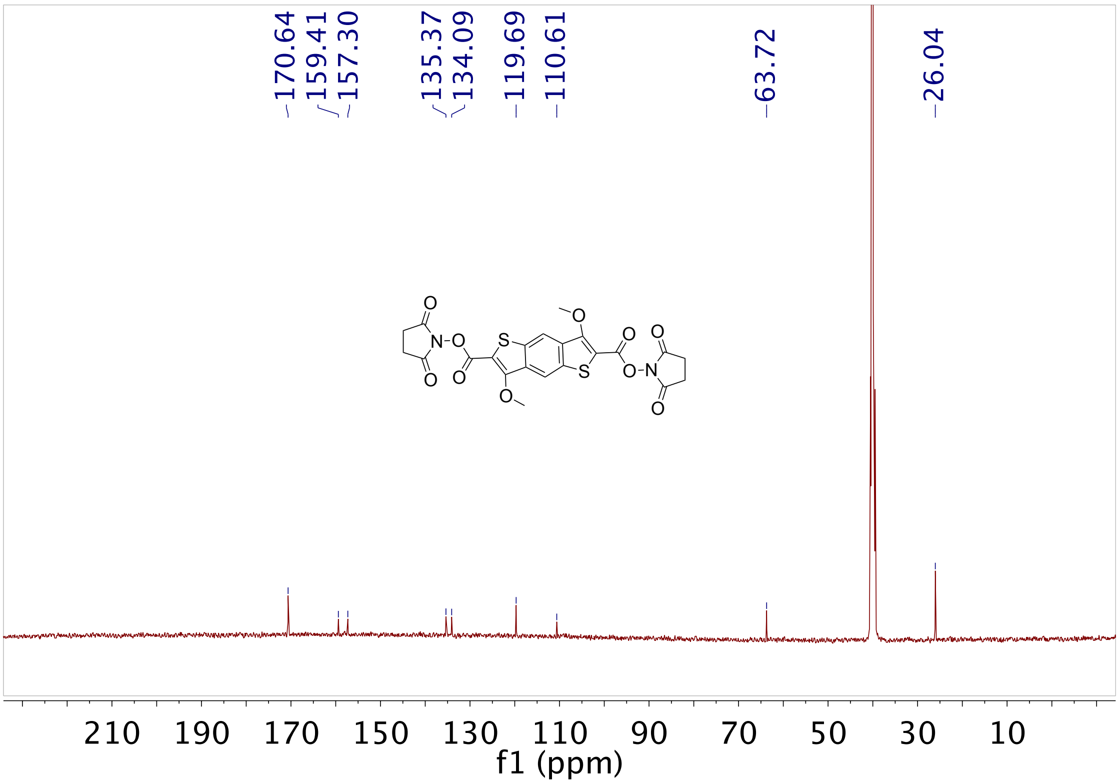

Supplementary Figure S6. ^{13}C NMR spectrum of **14** in DMSO-d_6.

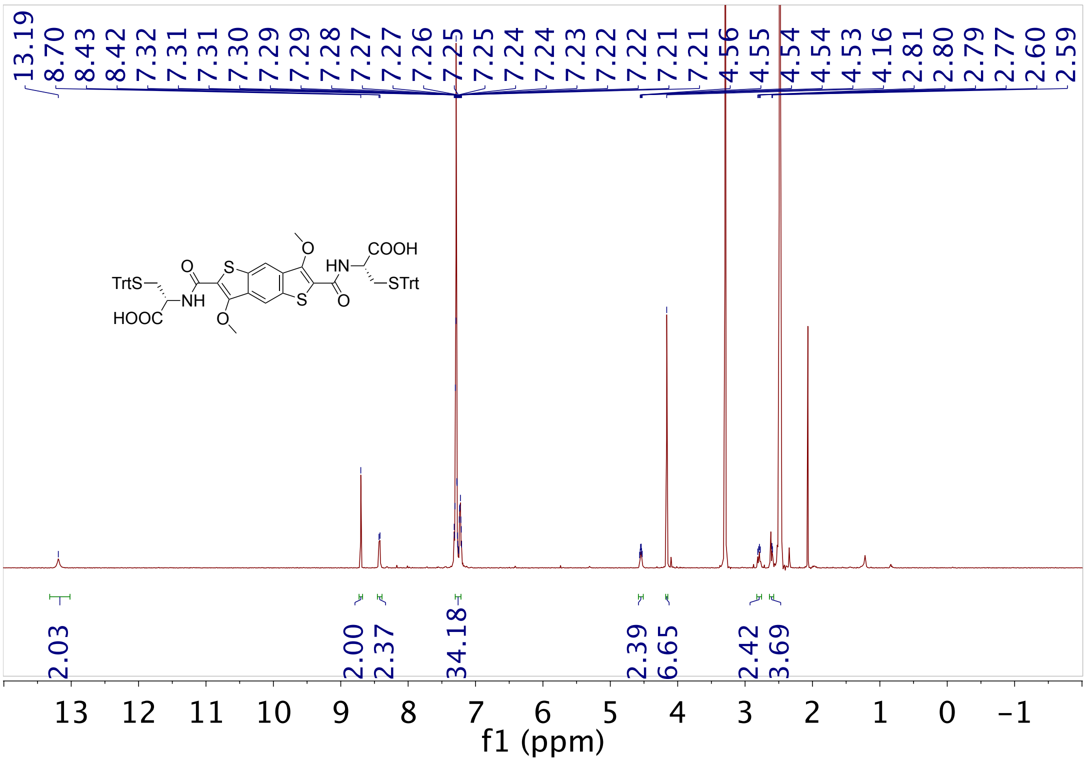

Supplementary Figure S7. ^{1}H NMR spectrum of **6** in DMSO-d_6.

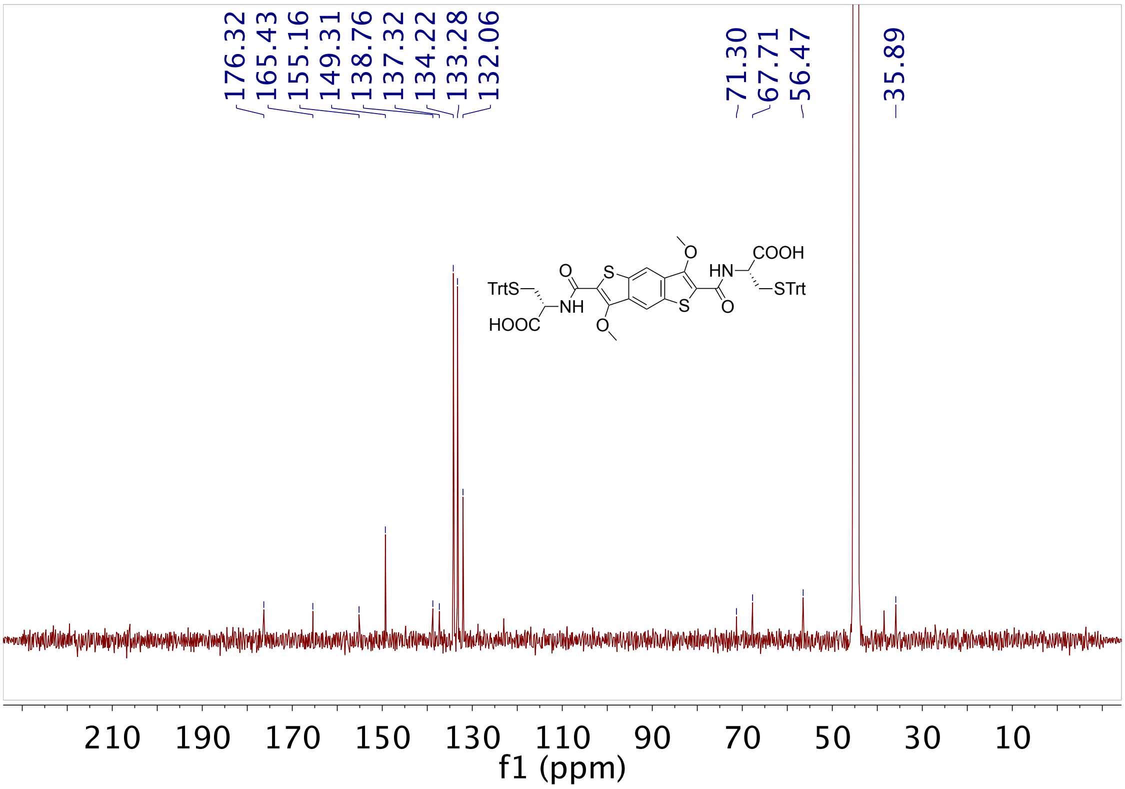

Supplementary Figure S8. ^{13}C NMR spectrum of **6** in DMSO-d_6.

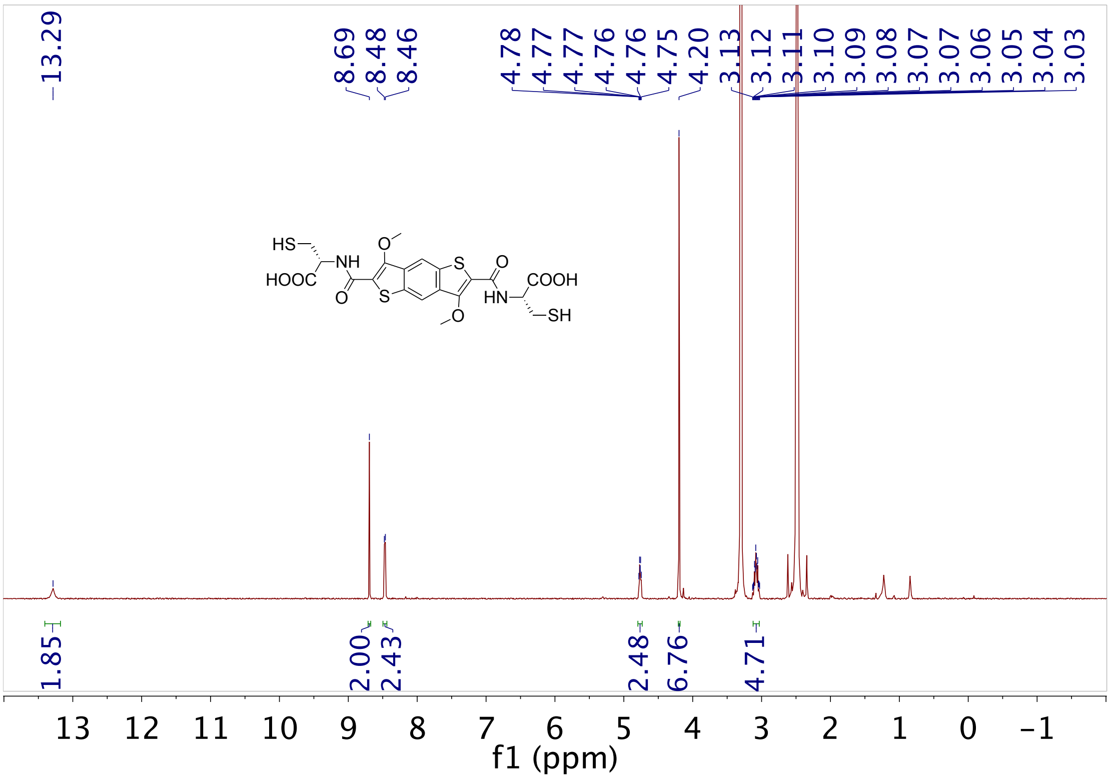

Supplementary Figure S9. ^{1}H NMR spectrum of **1** in DMSO-d_6.

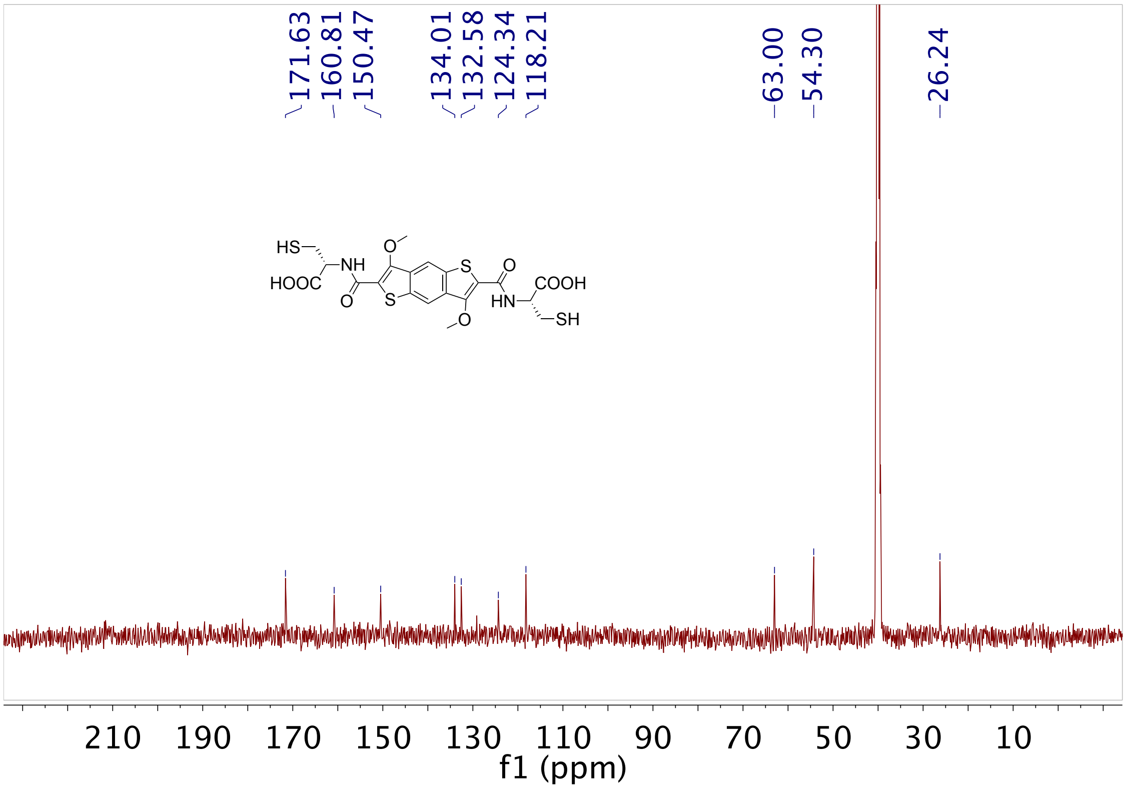

Supplementary Figure S10. ^{13}C NMR spectrum of **1** in DMSO-d_6.

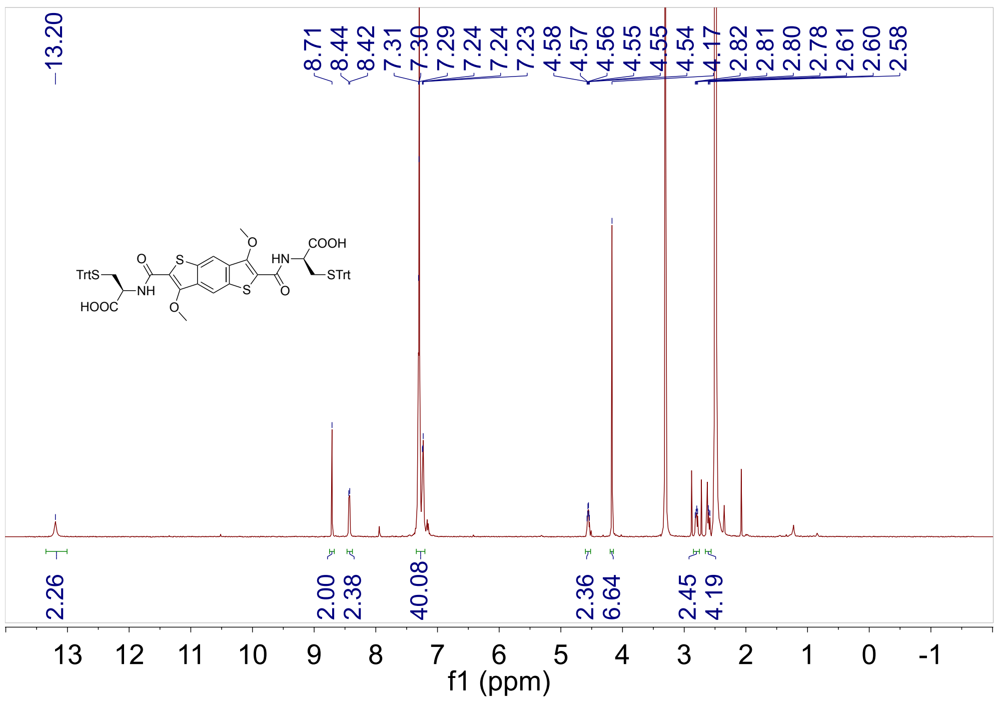

Supplementary Figure S11. ^{1}H NMR spectrum of **11** in DMSO-d_6.

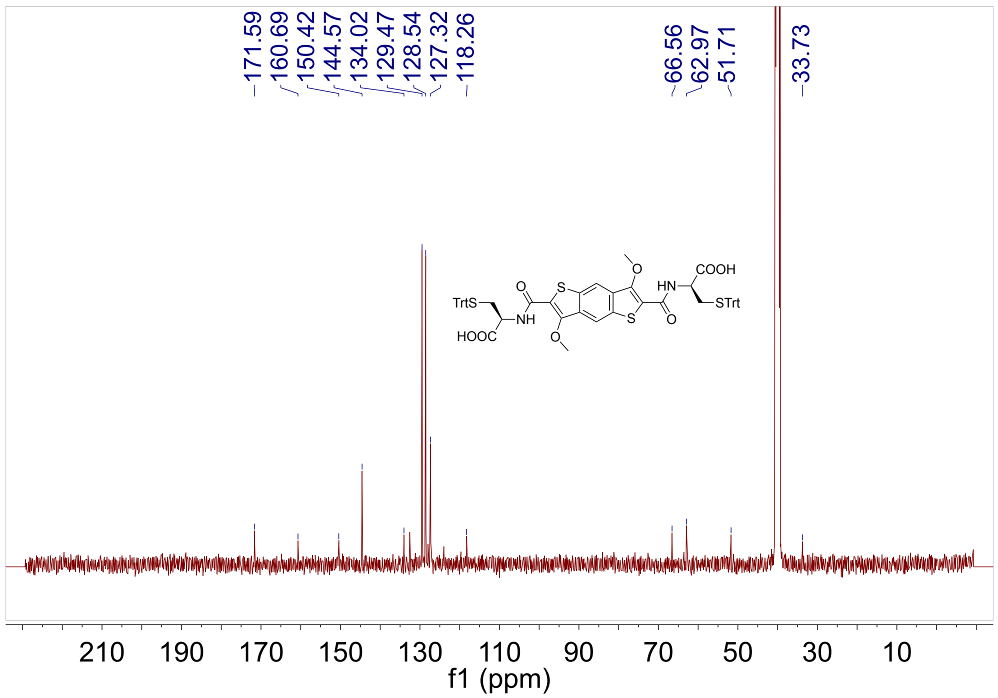

Supplementary Figure S12. ^{13}C NMR spectrum of **11** in DMSO-d_6.

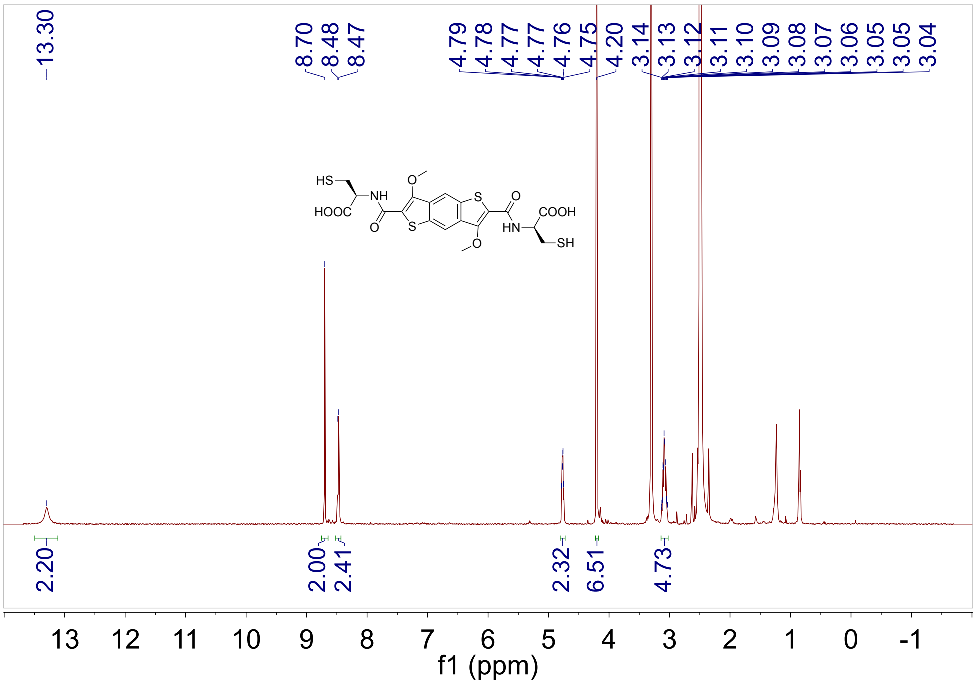

Supplementary Figure S13. ^{1}H NMR spectrum of **1*** in DMSO-d_6.

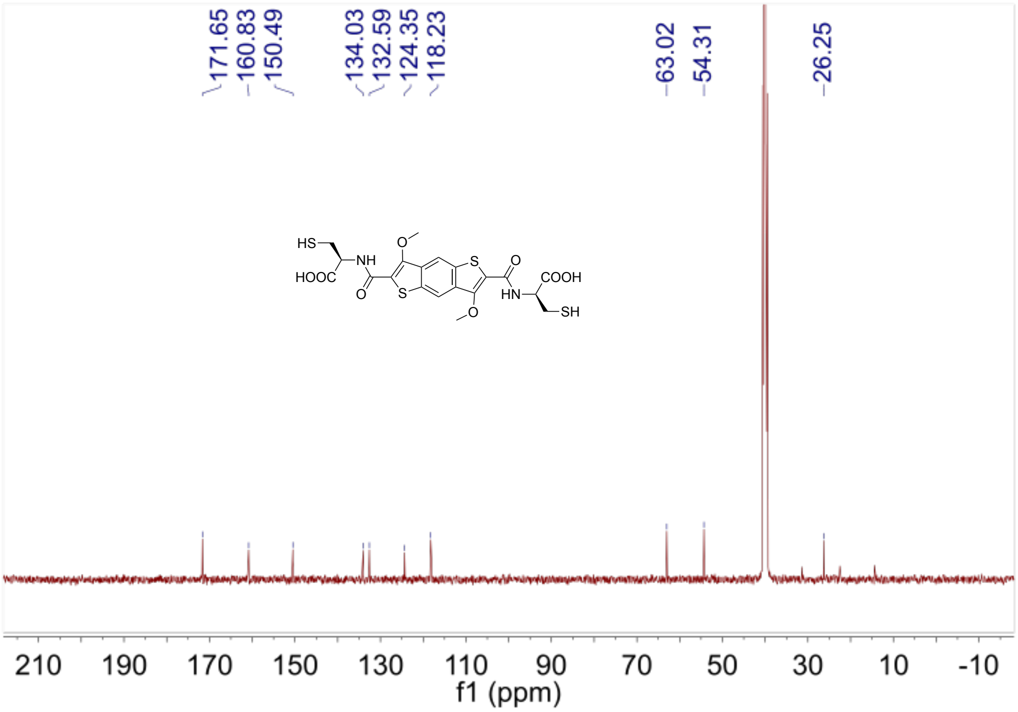

Supplementary Figure S14. ^{13}C NMR spectrum of **1*** in DMSO-d_6.

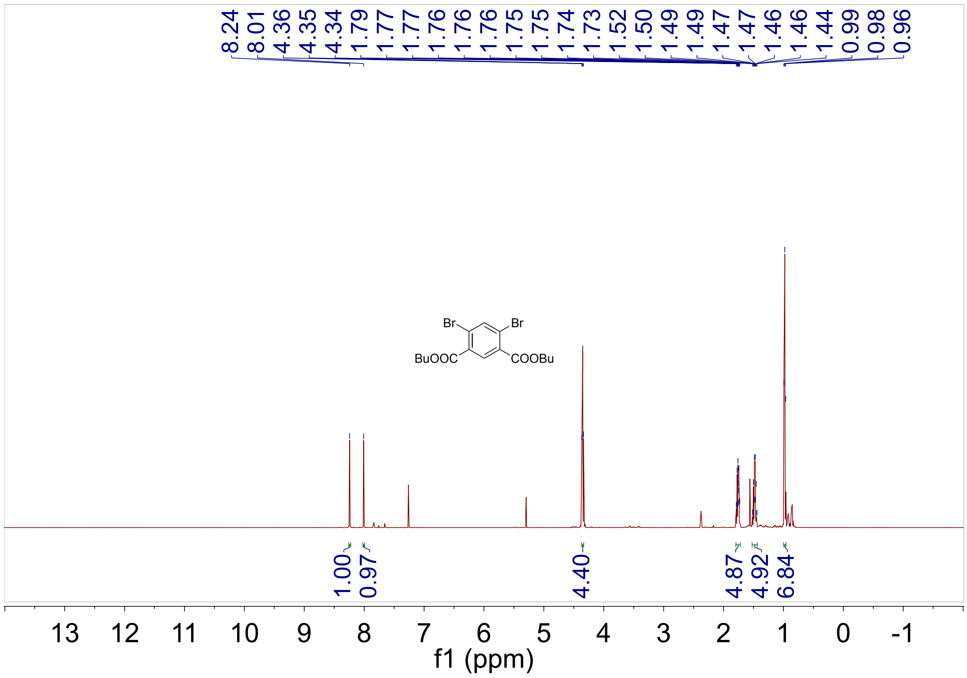

Supplementary Figure S15. ^{1}H NMR spectrum of **8** in $CDCl_3$.

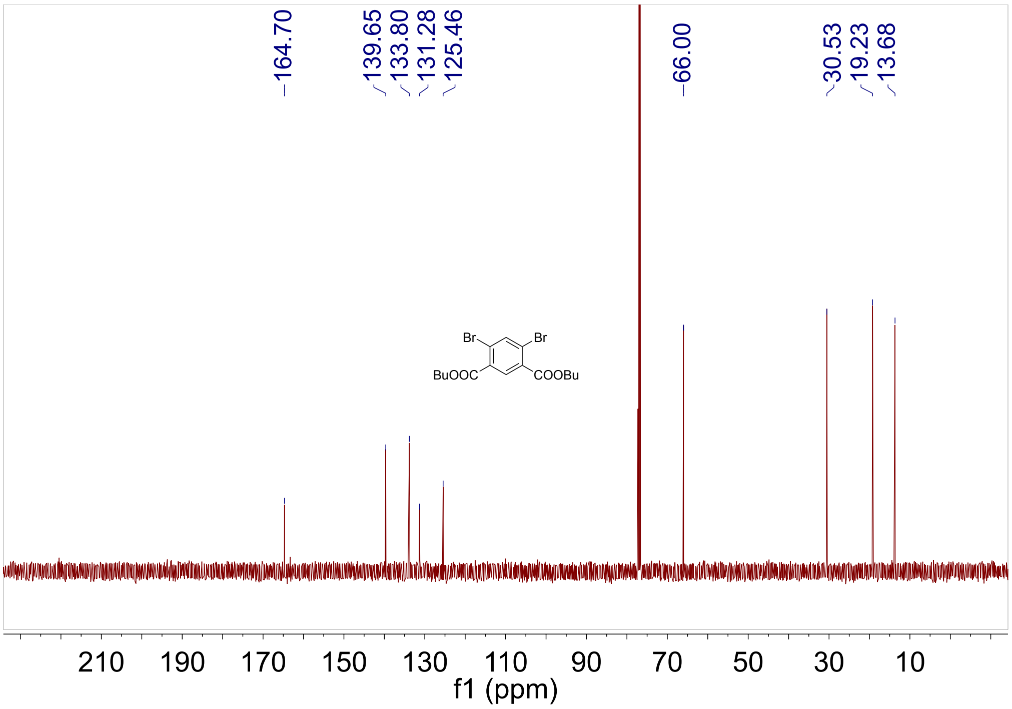

Supplementary Figure S16. ^{13}C NMR spectrum of **8** in $CDCl_3$.

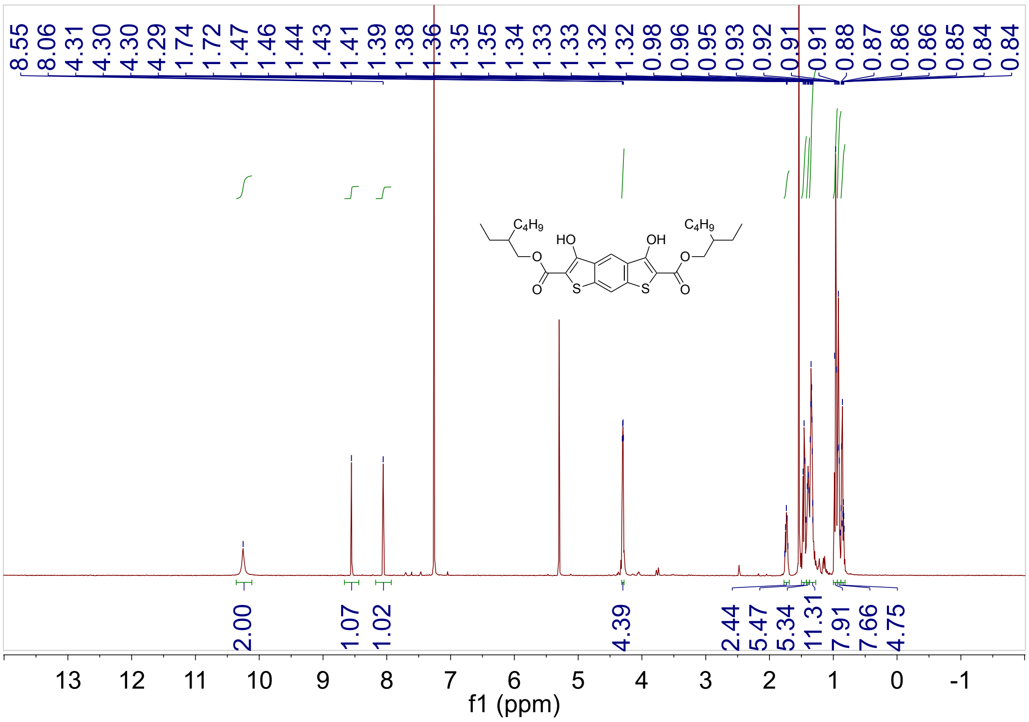

Supplementary Figure S17. ^{1}H NMR spectrum of **16** in $CDCl_3$.

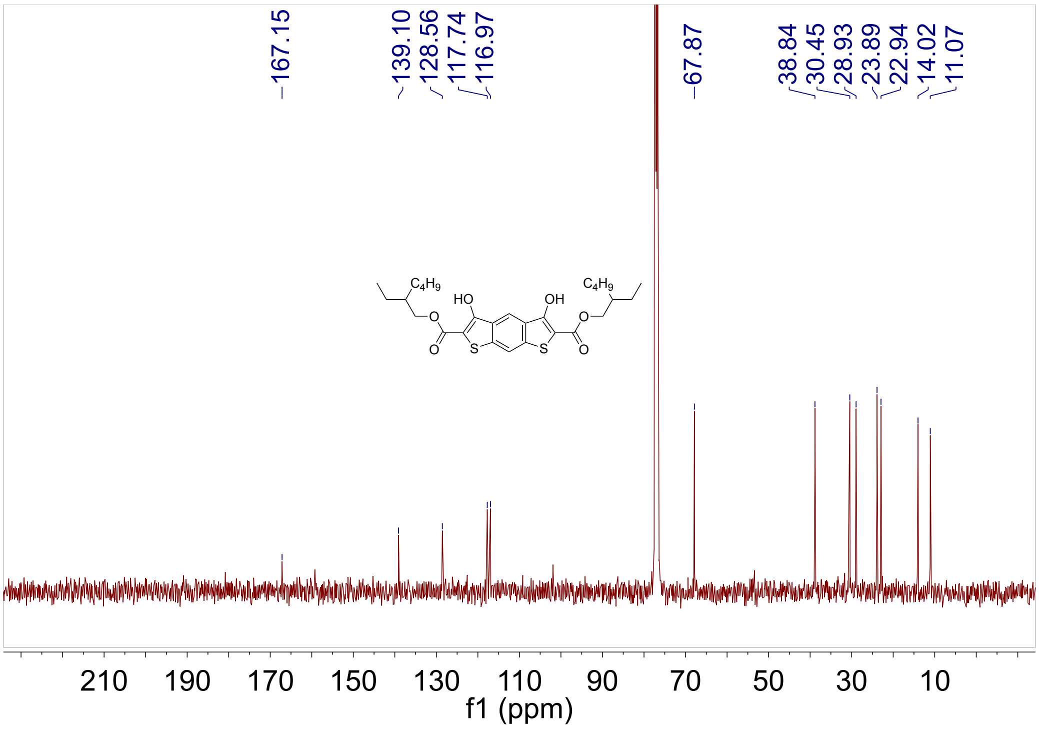

Supplementary Figure S18. ^{13}C NMR spectrum of **16** in $CDCl_3$.

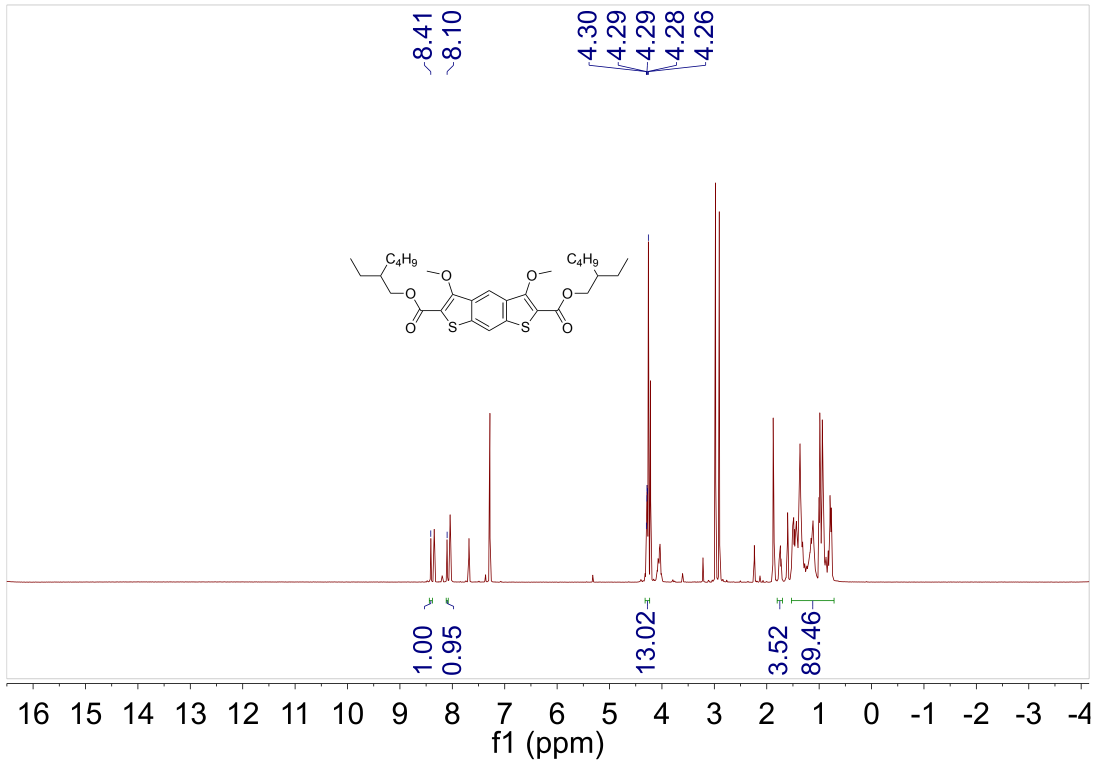

Supplementary Figure S19. ^{1}H NMR of spectrum **17** in $CDCl_3$.

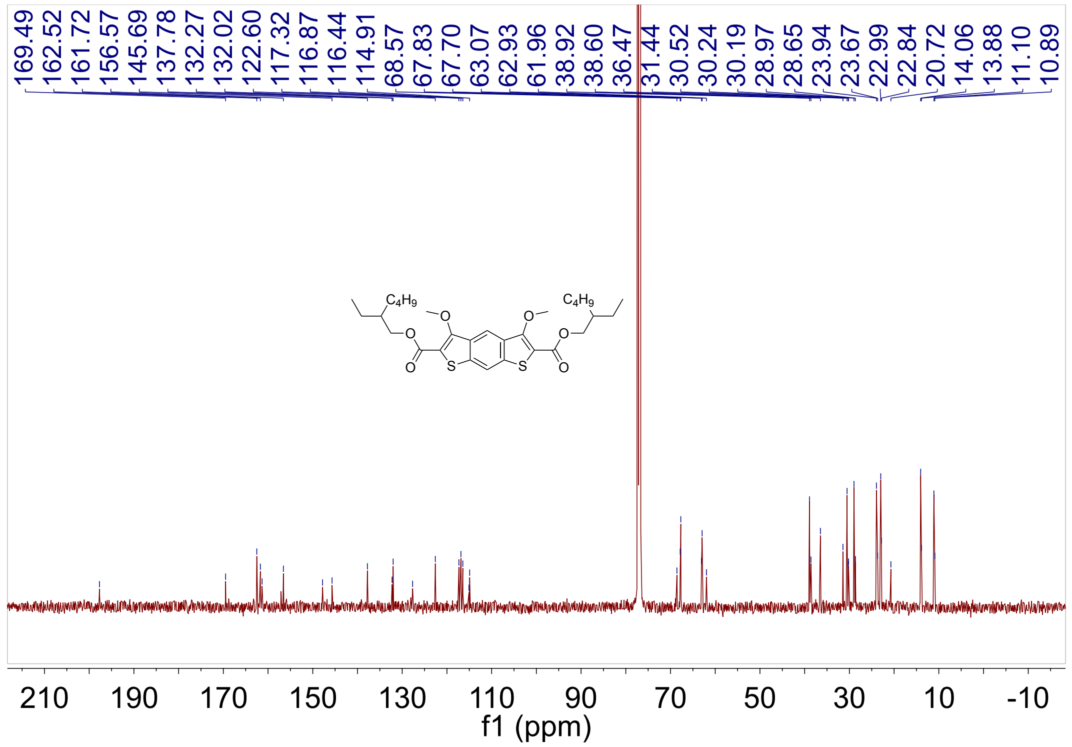

Supplementary Figure S20. ^{13}C NMR spectrum of **17** in $CDCl_3$.

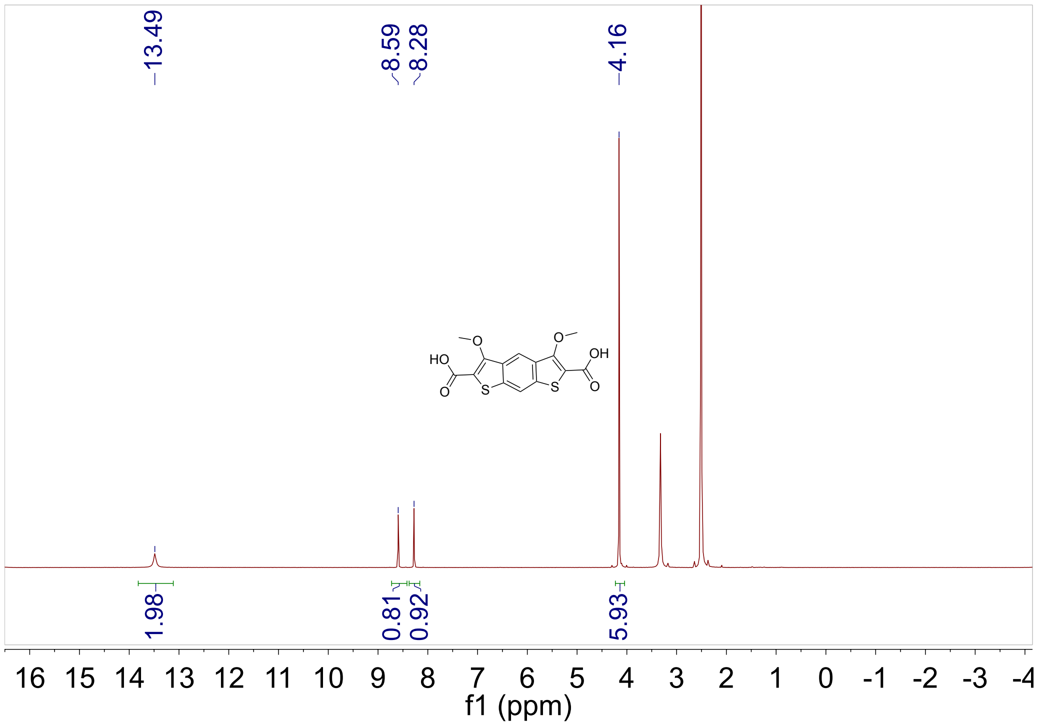

Supplementary Figure S21. ^{1}H NMR spectrum of **9** in DMSO-d_6.

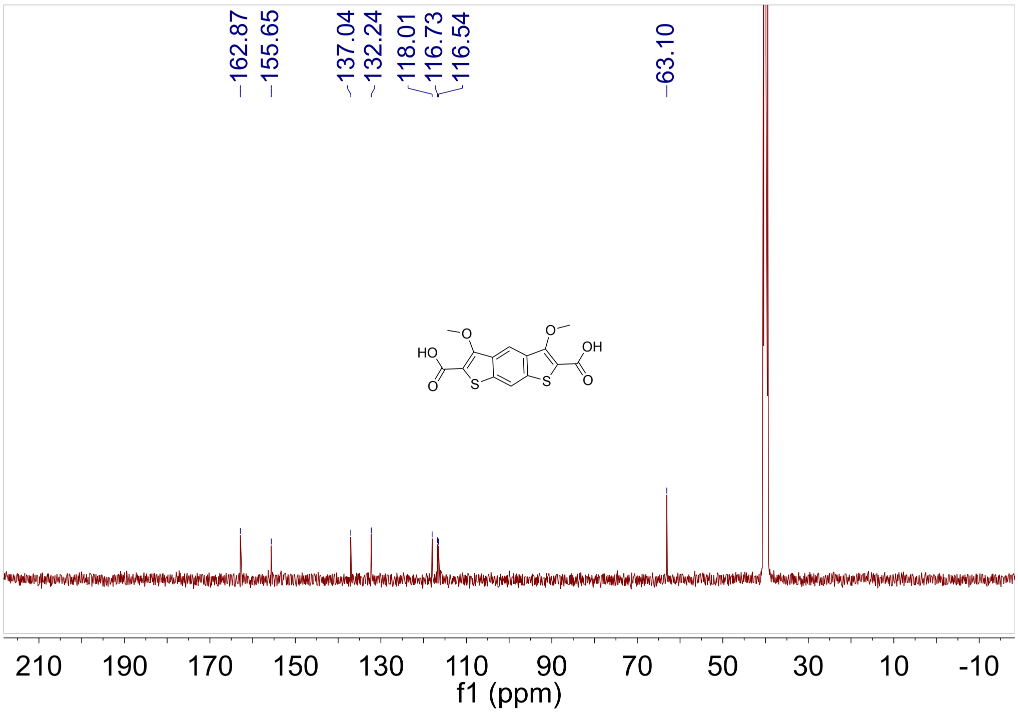

Supplementary Figure S22. ^{13}C NMR spectrum of **9** in DMSO-d_6.

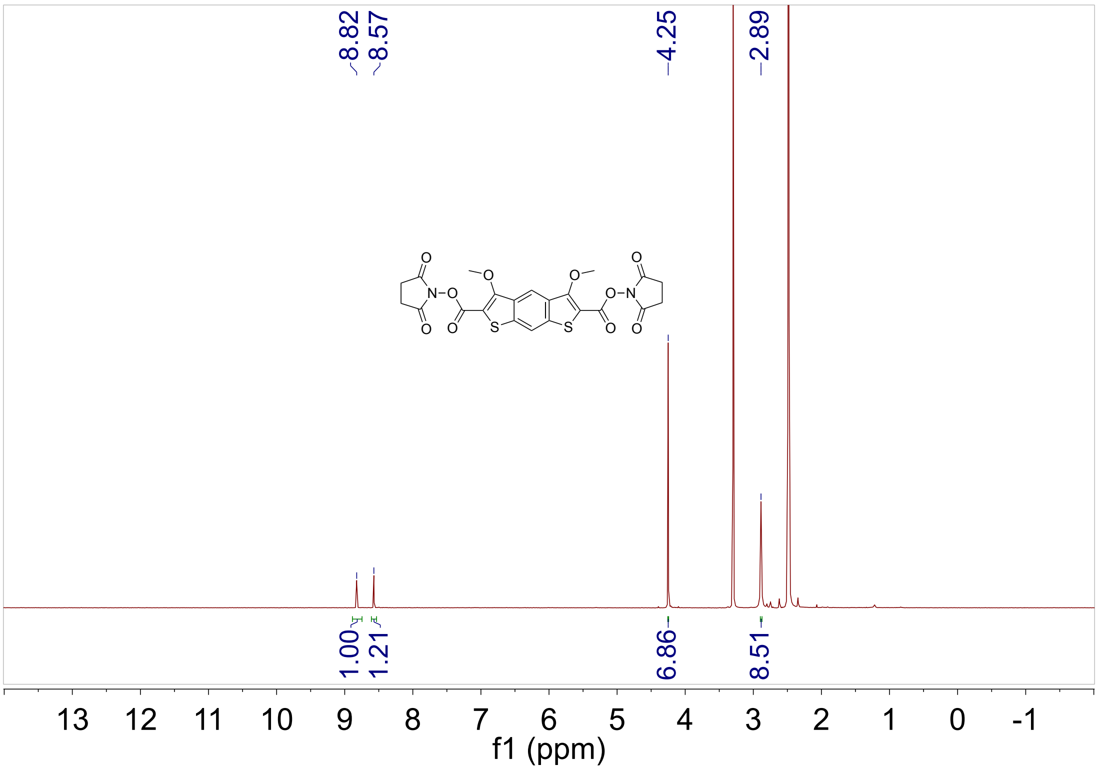

Supplementary Figure S23. ^{1}H NMR spectrum of 18 in DMSO-d_6.

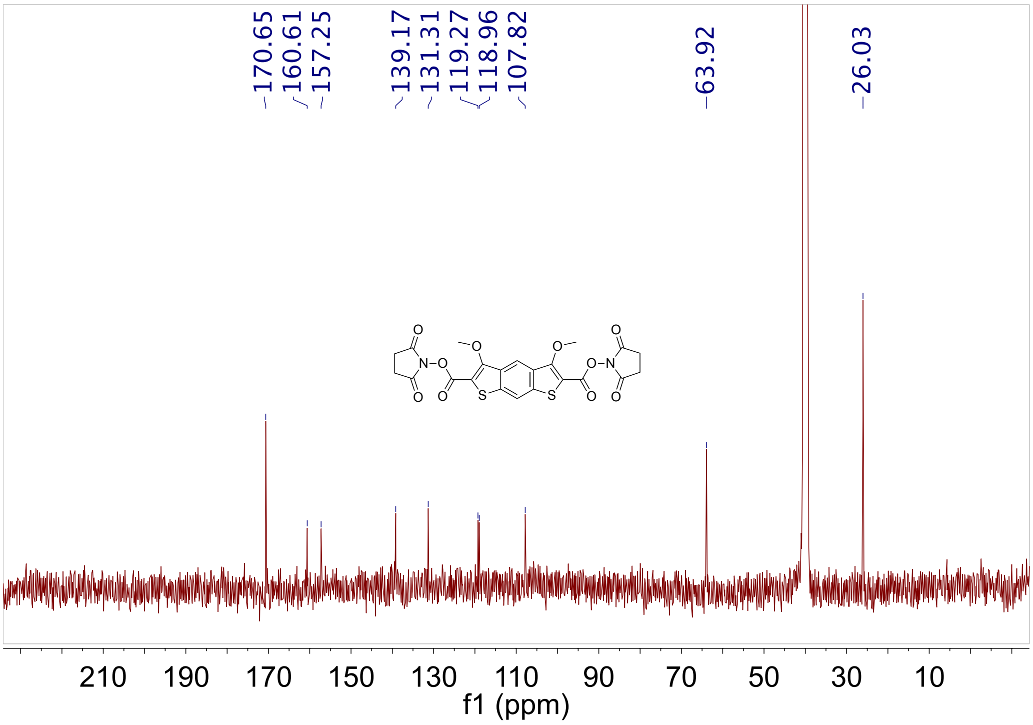

Supplementary Figure S24. ^{13}C NMR spectrum of 18 in DMSO-d_6.

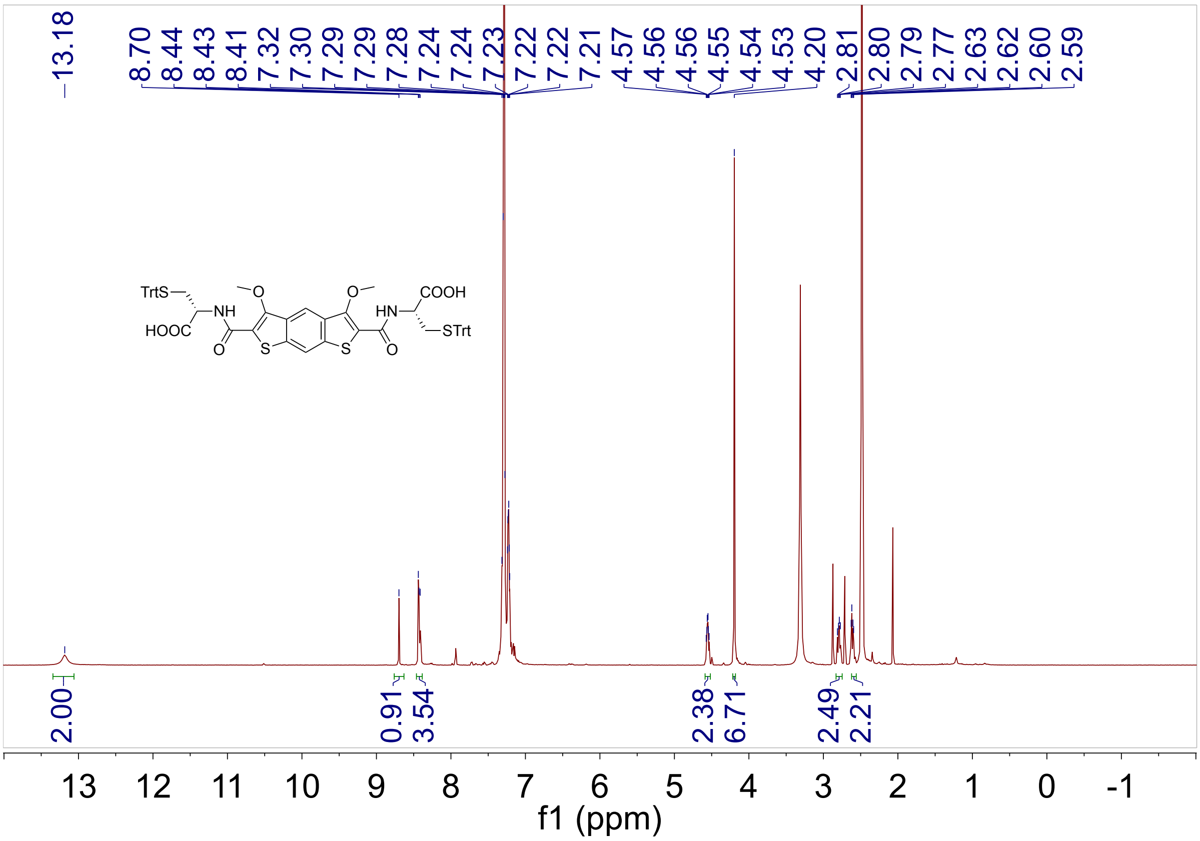

Supplementary Figure S25. ^{1}H NMR spectrum of **10** in DMSO-d_6.

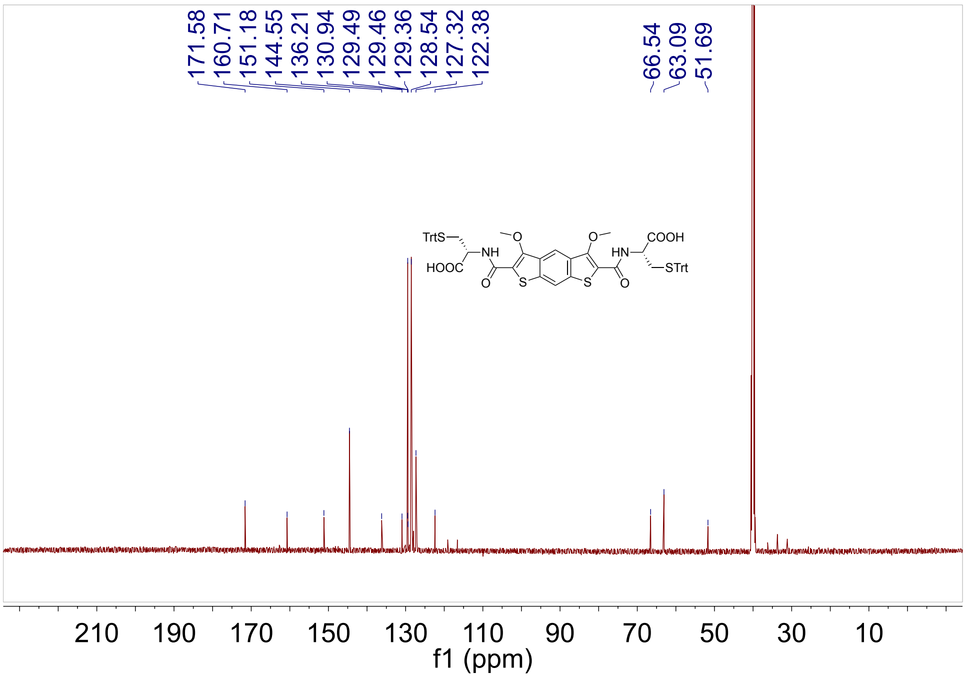

Supplementary Figure S26. ^{13}C NMR spectrum of **10** in DMSO-d_6.

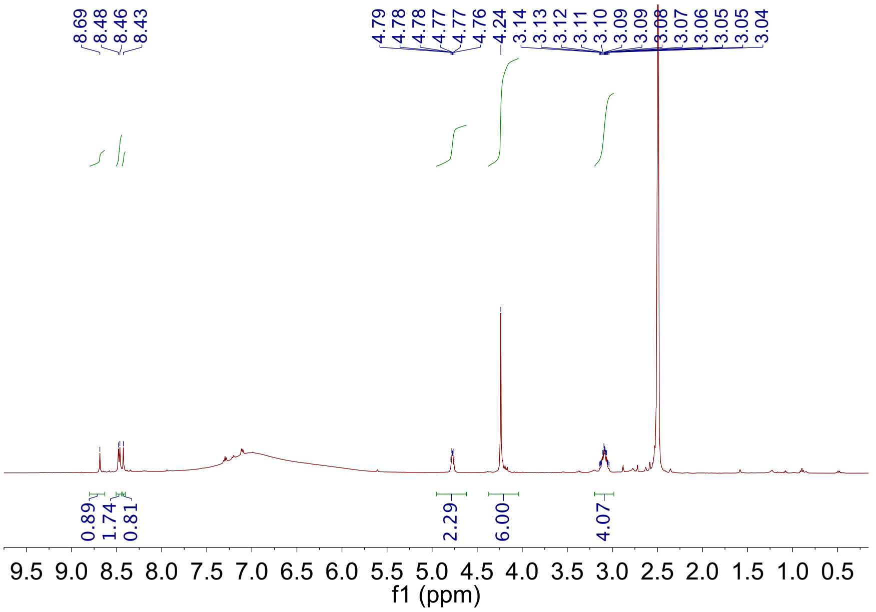

Supplementary Figure S27. ^{1}H NMR spectrum of **2** in DMSO-d_6 (the broad peak is TFA which was added to slow the thiol oxidation).

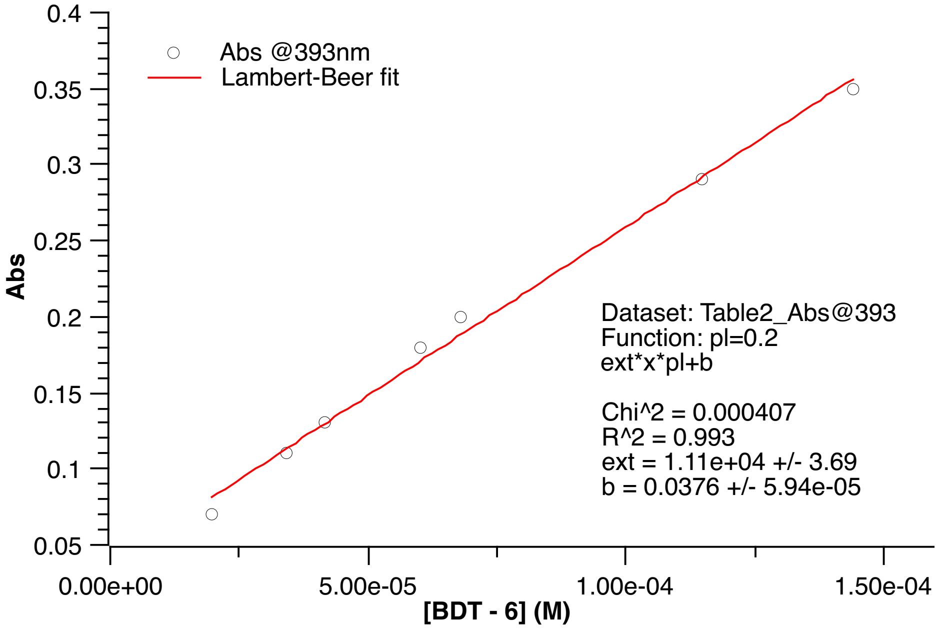

Supplementary Figure S28. Lambert-Beer plot for compound **6** at 393 nm.

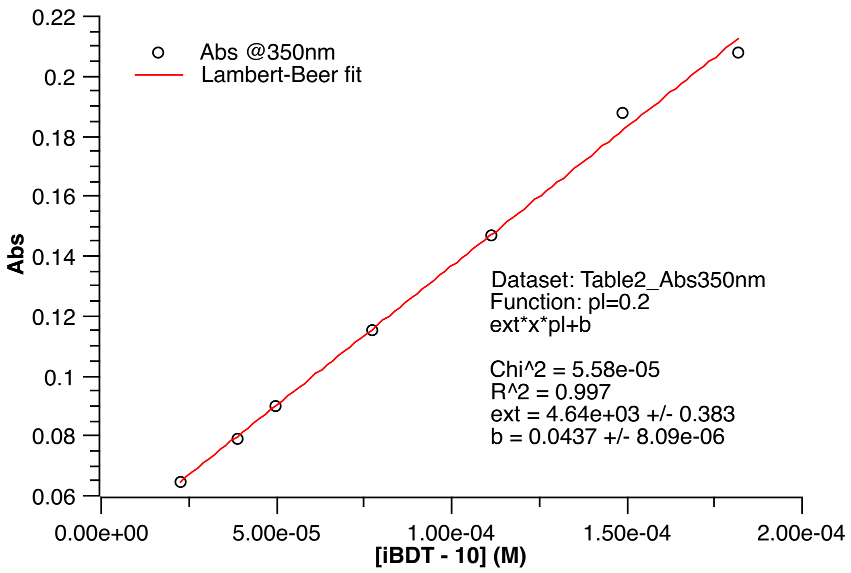

Supplementary Figure S29. Lambert-Beer plot for compound **10** at 350 nm.

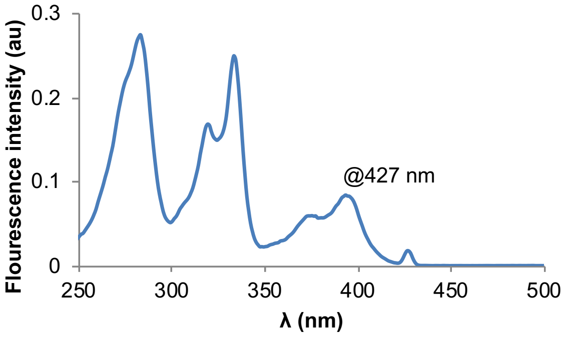

Supplementary Figure S30. The excitation spectrum of compound **6** (emission wavelength 427 nm).

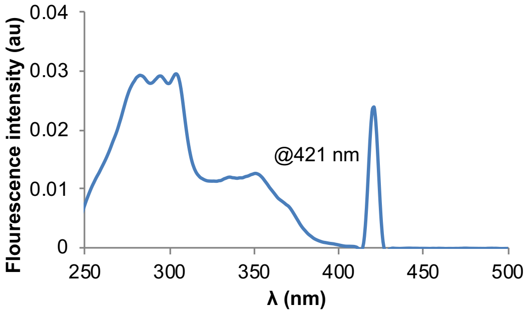

Supplementary Figure S31. The excitation spectrum of compound **10** (emission wavelength 421 nm).

DCL Set-up

A 5 mM library was prepared by dissolving the building block in 10 mM aqueous NaOH, followed by titration with 100 mM NaOH / 100 mM HCl to adjust the pH to 8. The DCL solutions were stirred in close-capped vials for at least 3 days (in the case of the catenane the stirring time was 1 day) and analysed by LC-MS. Preparative libraries (3 mL scale) were made using the same method as for the analytical libraries.

LC-MS Analysis of the DCLs

LC-MS Method (Thermo Surveyor PDA Plus LC and LCQ classic ESI MS)**:**

ESI-MS spectra (negative ion) were acquired with drying temperature of 250 °C, spray current 0.5 µA, sheath gas flow of 40 arb, spray voltage was set to 4.5 kV, capillary voltage 13 V, tube lens -15.0 V. The mass range was set from *m/z* 150-2000, the number of microscans in scan time was 5 and the maximum injection time was 150.0 ms.

MS/MS Setting (Thermo Surveyor PDA Plus LC and LCQ classic ESI MS)**:** Parent Mass (*m/z*): dependant on the species, Ionisation Width (*m/z*): 4.0, Normalized Collision Energy (%): 20, Activation Q: 0.250, Activation Time (msec) 30.0, the number of microscans in scan time was 5 and the maximum injection time was 150.0 ms.

HRMS Setting (Bruker MaXis HD ESI-QTOF)**:** ESI-MS spectra (negative ion) were acquired with drying temperature of 320 °C, collision energy -4 eV, dry gas 12 L/min. The mass range was set from m/z 350-3500.

MS/MS settings for HRMS data (Bruker MaXis HD ESI-QTOF): parent Mass (m/z): dependant on the species, ionisation width (m/z): 20.0, collision energy: 20 eV, drying temperature 240 °C, dry gas 12 L/min.

Analytical HPLC method for DCLs of 1 (5 mM): Column**:** Kromasil 100-5C8, 25 x 0.21 cm, 5 µm. Injection volume: 2 µL; Flow rate: 0.3 mL/min; Temperature: 39 °C; Run time: 10 min; Elution profile:

Method A:

Time / min	Water (0.1% FA)	CH_3CN (0.1% FA)
**0**	70%	30%
**10**	50%	50%

Preparative HPLC method for DCLs of all-donor L-[2]catenane (5 mM): Column: Kromasil 100-5C8, 25 x 0.8 cm, 5 µm; Injection volume: 500 µL; Flow rate: 3.5 mL/min; Temperature: 39 °C; Run time: 12.5 min; Elution profile:

Method B:

Time / min	Water (0.1% FA)	CH_3CN (0.1% FA)
**0**	70%	30%
**12.5**	50%	50%

Analytical HPLC method for DCLs of 1 with 1* (5 mM): Column: Kromasil C8 5u, 25 x 0.46 cm, 5 µm. Injection volume: 4 µL; Flow rate: 1 mL/min; Temperature: 39 °C; Run time: 37 min; Elution profile:

Method C:

Time / min	Water (0.1% FA)	CH_3CN (0.1% FA)
**0**	90%	10%
**20**	70%	30%
**35**	65%	35%
**37**	0%	100%

Analytical HPLC method for DCLs of all libraries with 2 (5 mM): Column: Ultra BiPhenyl, 5 x 0.3 cm, 3 µm. Injection volume: 3 µL; Flow rate: 0.5 mL/min; Temperature: 39 °C; Run time: 19 min; Elution profile:

Method D:

Time / min	Water (0.1% FA)	CH_3OH (0.1% FA)
**0**	50%	50%
**10**	5%	95%
**14**	5%	95%
**14.1**	50%	50%
**19**	50%	50%

All-donor [2]catenane synthesis:

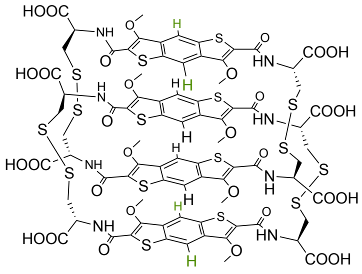

A 5 mM library of **1** was prepared (3 mL) was prepared by dissolving **1** in 10 mM NaOH then pH was adjusted to 8 adding aliquots of 100 mM HCl / 100 mM NaOH. After 1 day of stirring, the library was fully oxidised and the catenane was isolated using semi-preparative HPLC (Method B). ^{1}H NMR (500 MHz, D_2O): δ 7.67 (s, 4H), 6.69 (s, 4H), 4.08 (s, 12H), 3.97 (s, 2H), 3.67 (dd, *J* = 15.0, 4.4 Hz, 4H), 3.57 – 3.51 (m, 6H), 3.49 – 3.42 (m, 7H). The α protons are under the solvent peaks and because the experiment was run under solvent suppression conditions they can't be seen. The β protons integration gives 17 protons instead of 16 protons due a small impurity of **1 homodimer** from HPLC separation (the dimer was dragging on the column).^{13}C NMR was not acquired due to insufficient material. HRMS ESI: *m/z* calcd for $C_{80}H_{72}N_8O_{32}S_{16}$ $[M-2H]^{2-}$ 1082.9808, found 1082.9854.

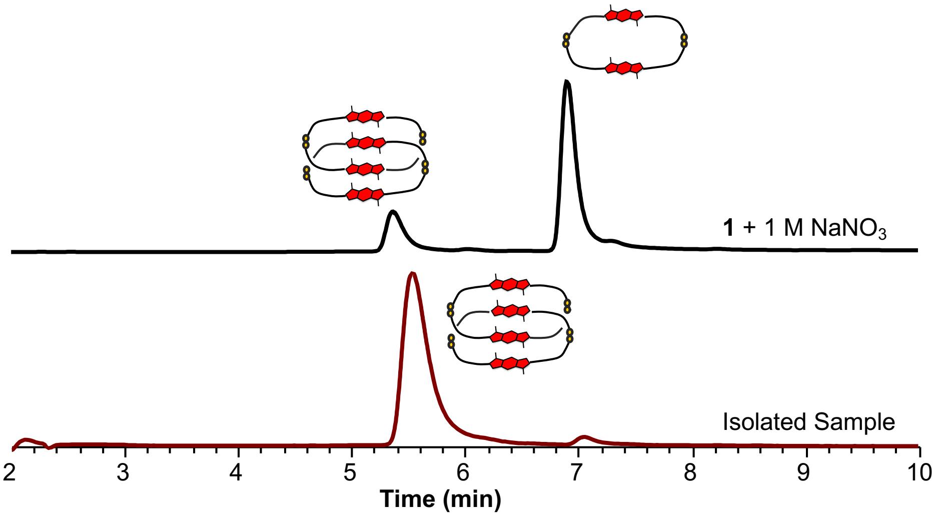

Supplementary Figure S32. Reverse-phase HPLC analysis of **1** (5 mM concentration) library in the presence of 1 M $NaNO_3$ and b) the reverse-phase HPLC analysis of the isolated **all-donor L-[2]catenane**. Absorbance was recorded at 391 nm.

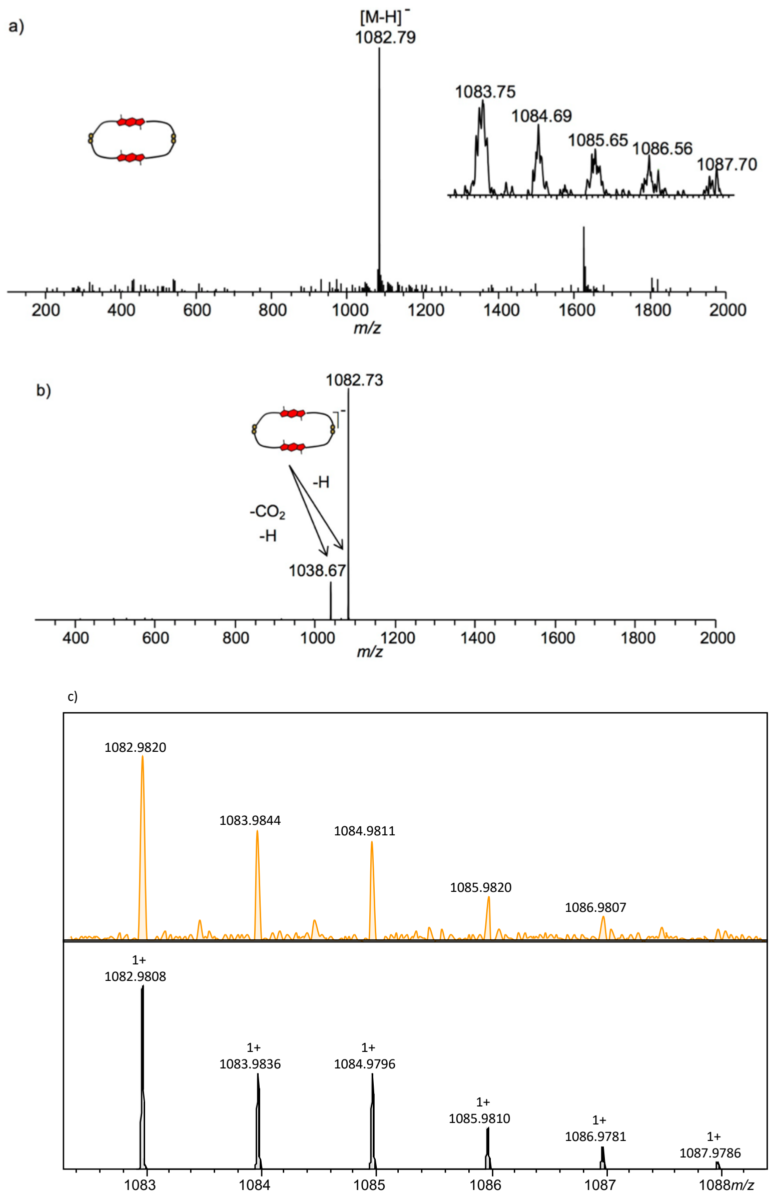

Supplementary Figure S33. a) MS (-*ve*) of **1 homodimer**; zoom of molecular ion is shown as inset. b) MS/MS (-*ve*) of **1 homodimer**; c) HRMS of catenane (top) and the simulated isotope pattern (bottom).

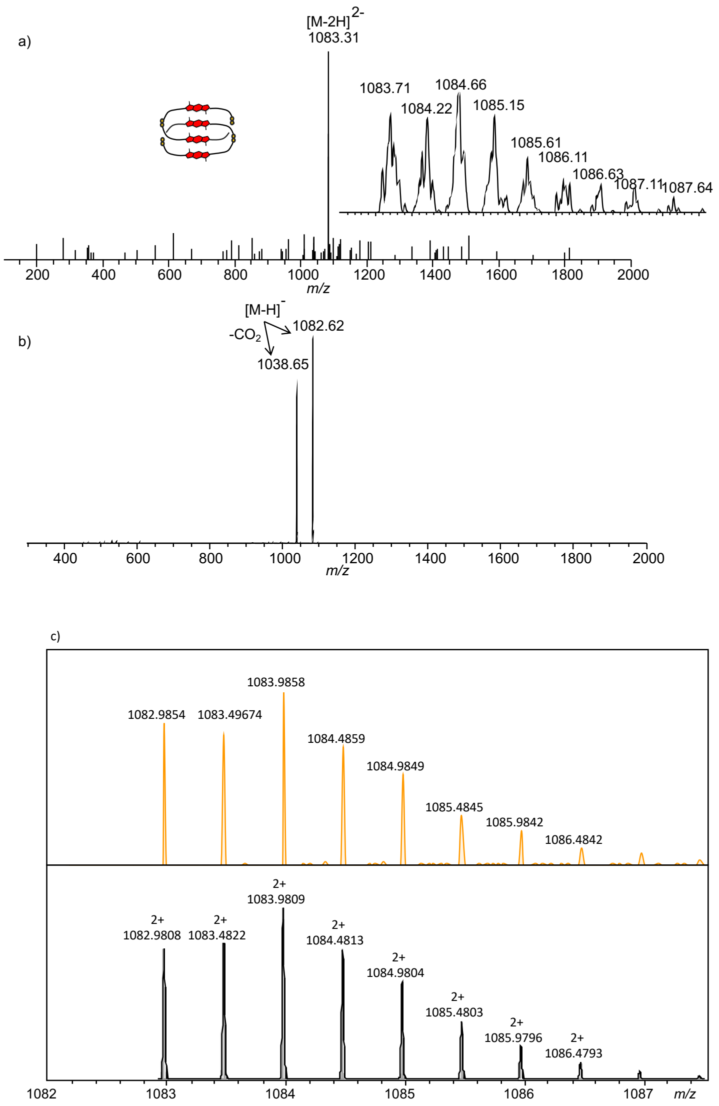

Supplementary Figure S34. a) MS (-*ve*) of **all-donor L-[2]catenane**; zoom of molecular ion is shown as inset; b) MS/MS (-*ve*) of **all-donor L-[2]catenane**; c) HRMS of catenane (top) and the simulated isotope pattern (bottom).

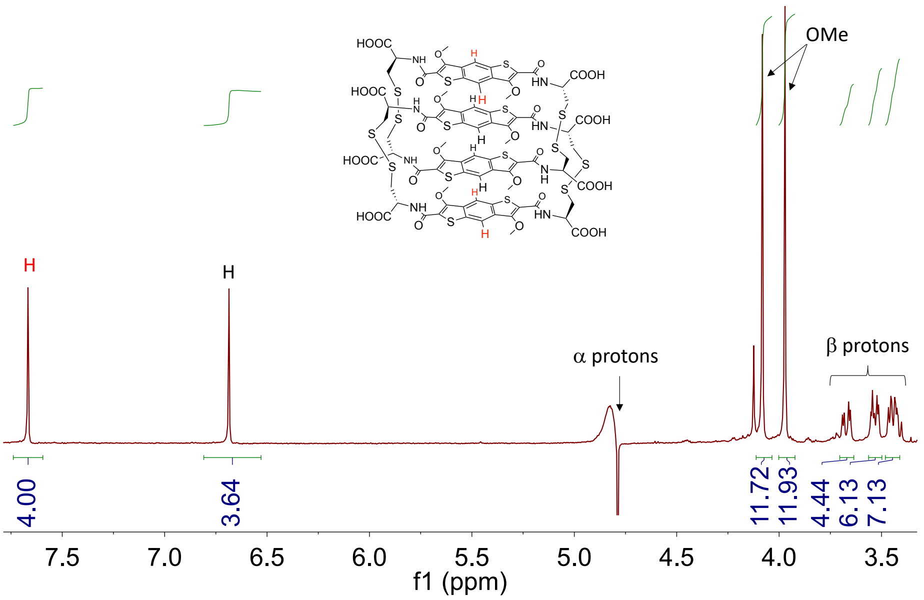

Supplementary Figure S35. ^{1}H NMR spectrum of all-donor [2]catenane in D_2O.

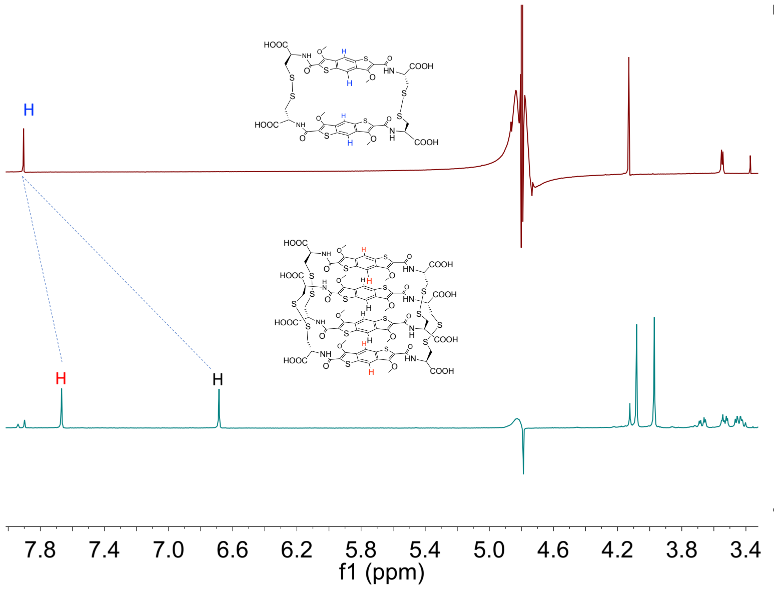

Supplementary Figure S36. ^{1}H NMR spectrum of dimer in H_2O/D_2O and all-donor [2]catenane in D_2O.

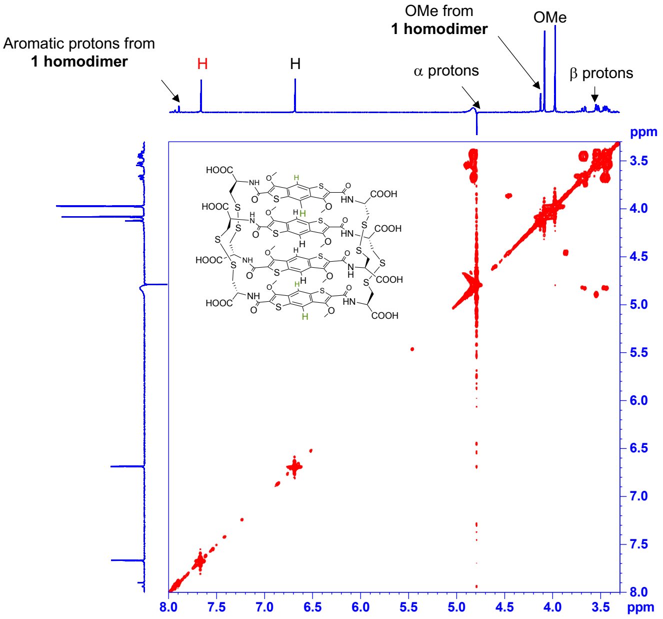

Supplementary Figure S37. ^{1}H-^{1}H COSY spectra (500 MHz, 298 K) of **all-donor L-[2]catenane**. The solvent (H_2O) was referenced at 4.79 ppm.

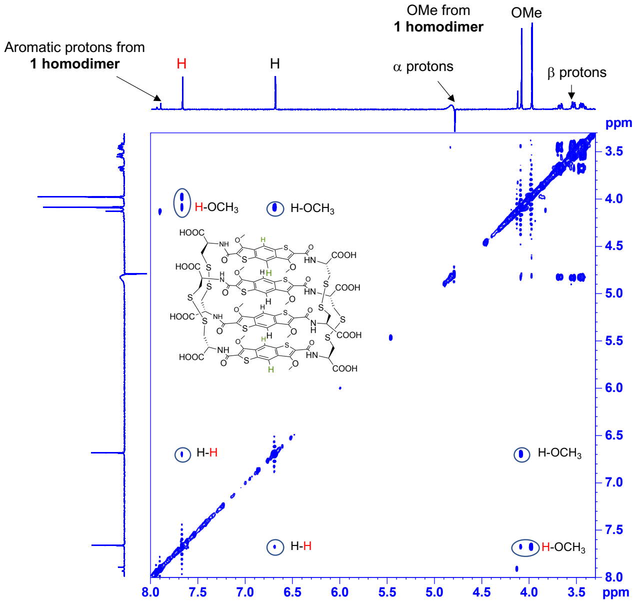

Supplementary Figure S38. 2D NOESY spectrum (500 MHz, 298 K) of **all-donor L-[2]catenane**. The solvent (H_2O) was referenced at 4.79 ppm.

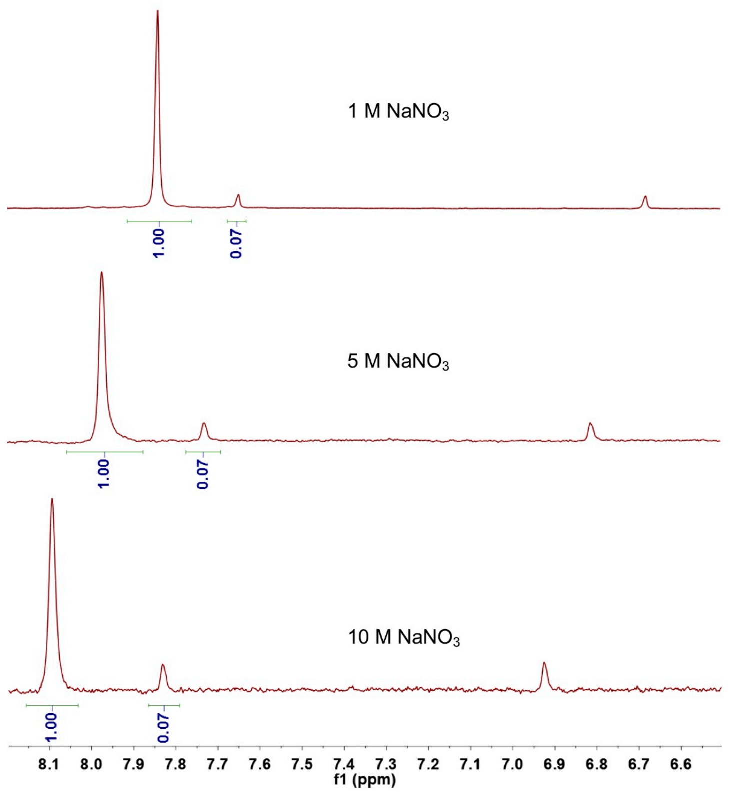

Supplementary Figure S39. The ^{1}H NMR spectra of **1** (5 mM concentration) libraries in the presence of 1 M $NaNO_3$, 5 M $NaNO_3$ and 10 M $NaNO_3$.

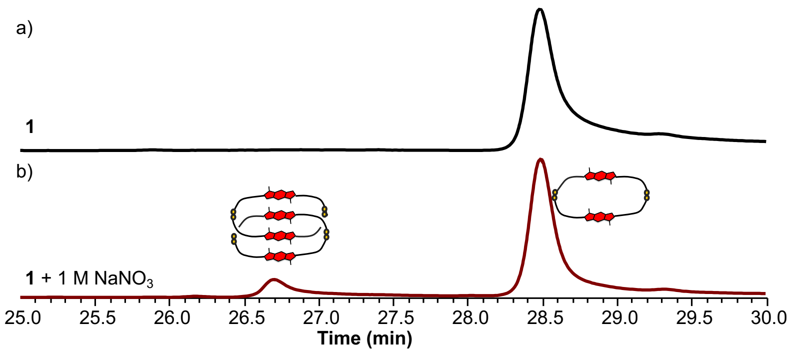

Supplementary Figure S40. Reverse-phase HPLC analysis of **1** (5 mM concentration) library a) without salt and b) in the presence of 1 M $NaNO_3$. Absorbance was recorded at 391 nm.

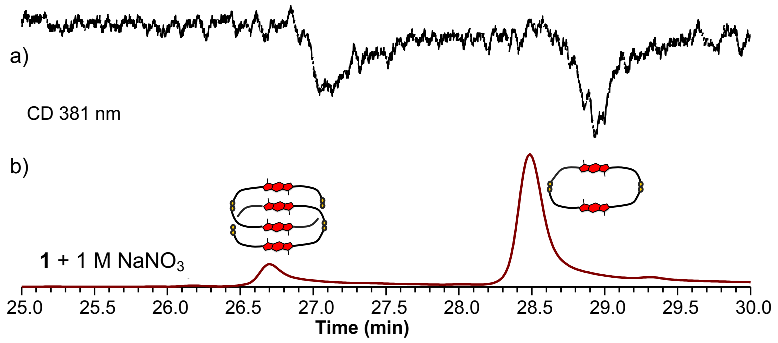

Supplementary Figure S41. Reverse-phase HPLC analysis of **1** (5 mM concentration) library in the presence of 1 M $NaNO_3$ showing a) CD at 381 nm and b) absorbance at 391 nm.

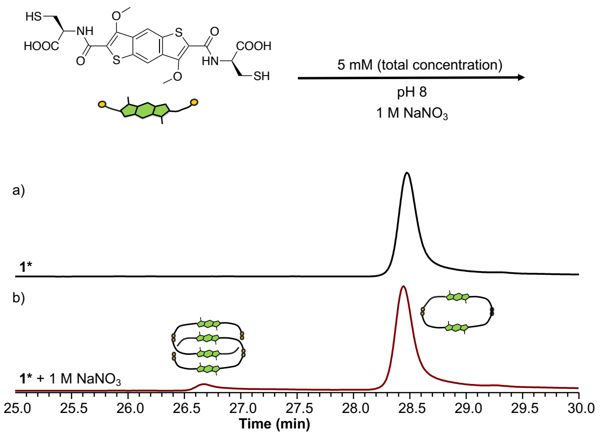

Supplementary Figure S42. a) Reverse-phase HPLC analysis of **1*** (5 mM concentration) library without salt and b) in the presence of 1 M $NaNO_3$. Absorbance was recorded at 391 nm.

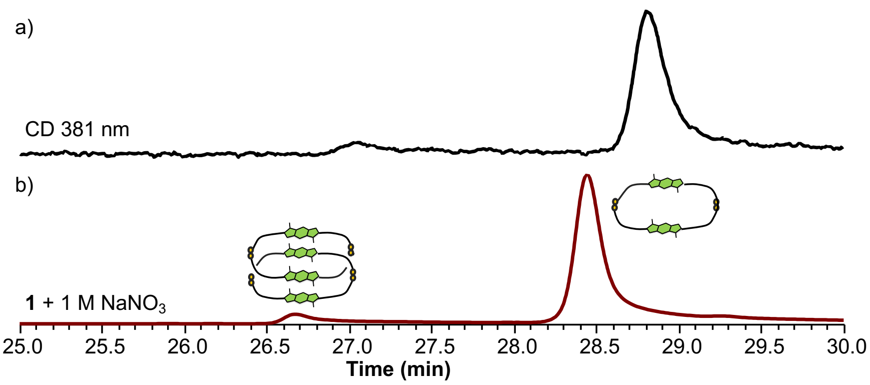

Supplementary Figure S43. Reverse-phase HPLC analysis of **1*** (5 mM concentration) library in the presence of 1 M $NaNO_3$ showing a) CD at 381 nm and b) absorbance at 391 nm.

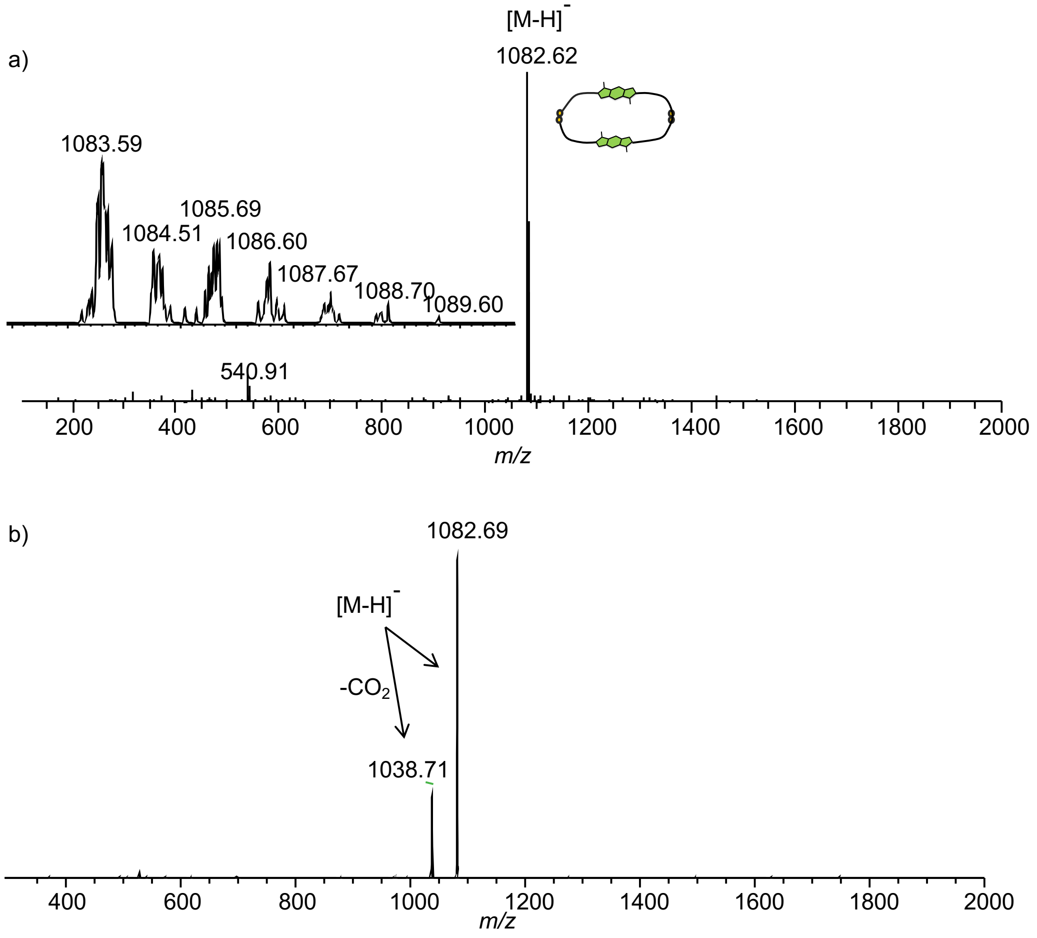

Supplementary Figure S44. a) MS (*-ve*) of **1* homodimer**; zoom of the molecular ion is shown as an inset and b) MS/MS (*-ve*) of **1* homodimer**.

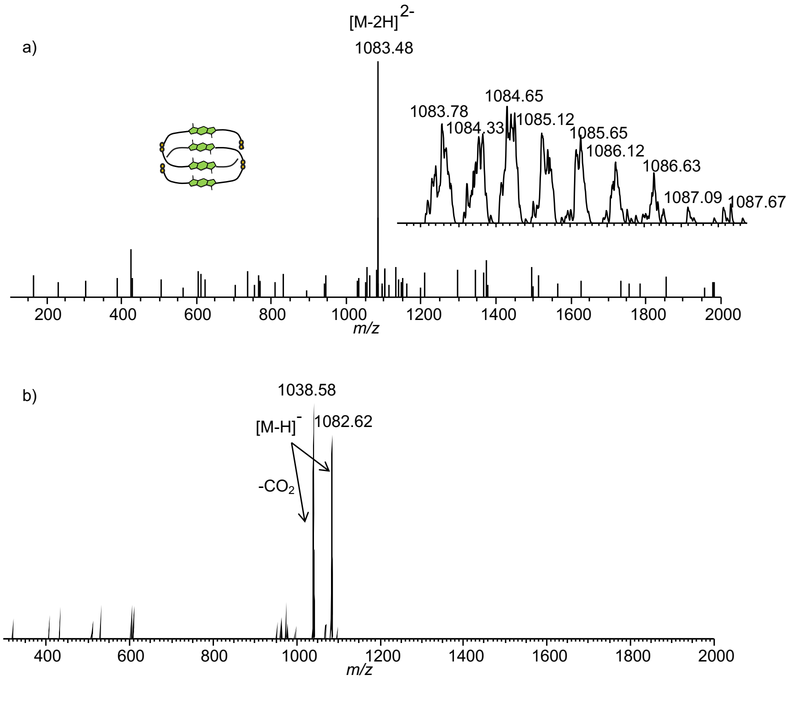

Supplementary Figure S45. a) MS (*-ve*) of **all-donor D-[2]catenane**; zoom of the molecular ion is shown as an inset and b) MS/MS *(-ve)* of **all-donor D-[2]catenane**.

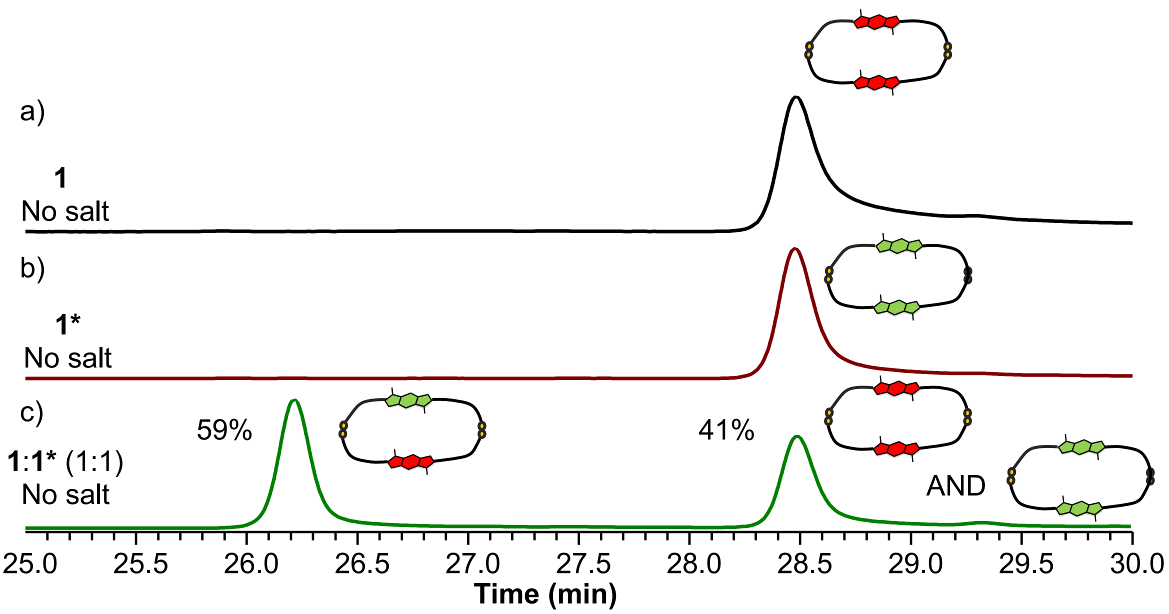

Supplementary Figure S46. a) Reverse-phase HPLC analysis of **1** (5 mM concentration), b) **1*** (5 mM concentration) and c) **1:1*** (1:1 molar ratio, 5 mM concentration) libraries without salt. Absorbance was recorded at 391 nm.

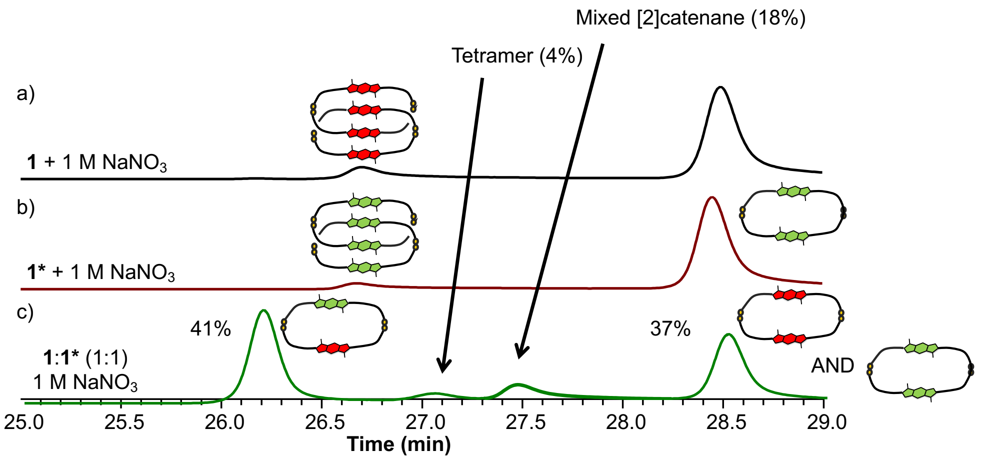

Supplementary Figure S47. a) Reverse-phase HPLC analysis of **1** (5 mM concentration), b) **1*** (5 mM concentration) and c) **1:1*** (1:1 molar ratio, 5 mM concentration) libraries in the presence of 1 M $NaNO_3$. Absorbance was recorded at 391 nm.

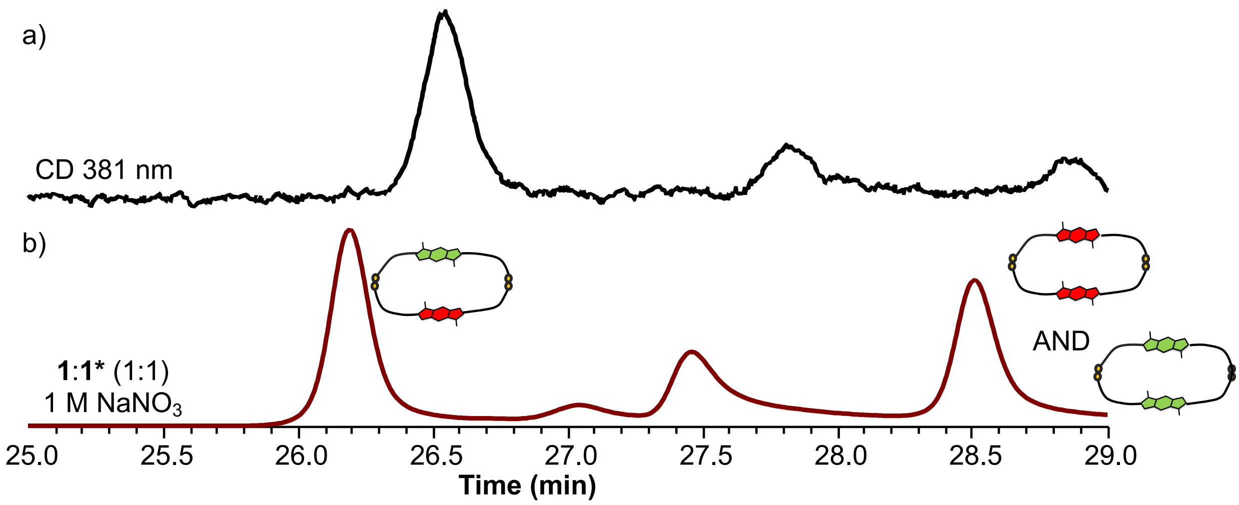

Supplementary Figure S48. Reverse-phase HPLC analysis of **1**:**1*** (1:1 molar ratio, 5 mM concentration) library in the presence of 1 M $NaNO_3$ showing a) CD at 381 nm and b) absorbance at 381 nm

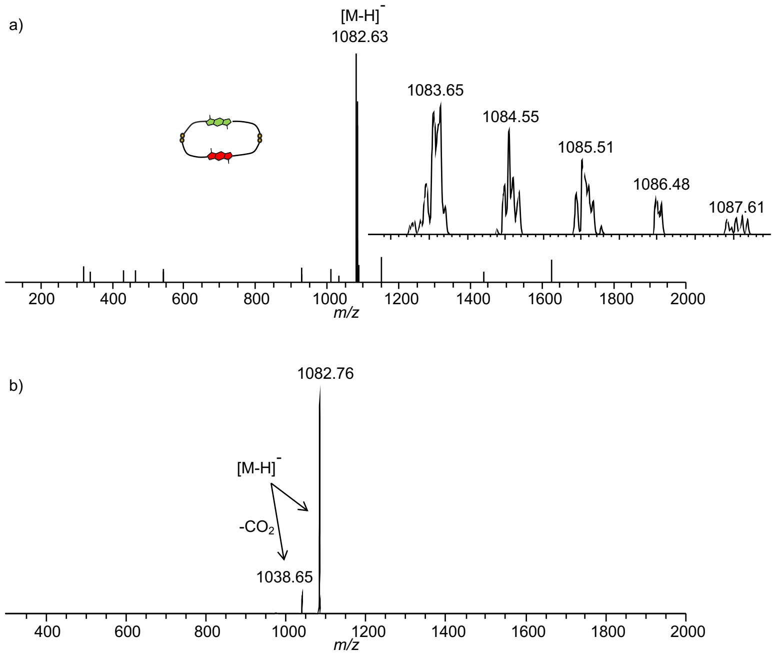

Supplementary Figure S49. a) MS (-*ve*) of **heterochiral homodimer**; zoom of the molecular ion is shown as an inset and b) MS/MS (-*ve*) of **heterochiral homodimer**.

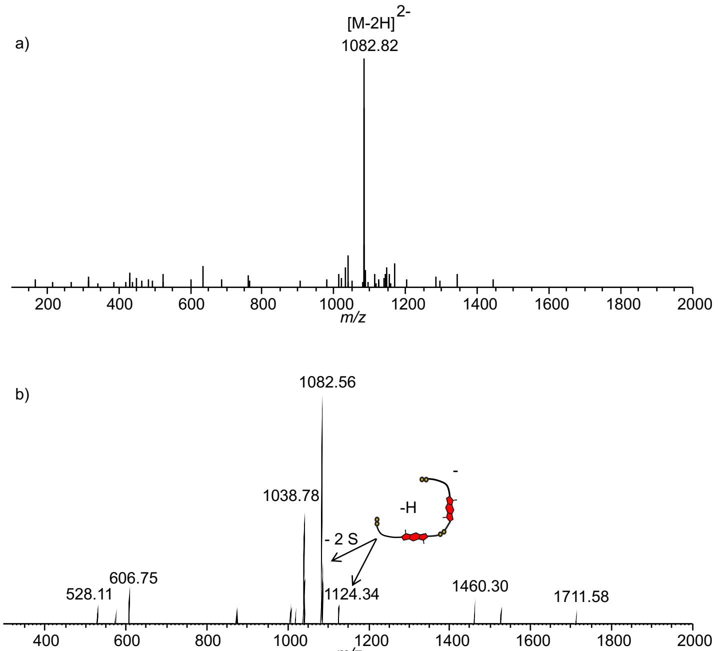

Supplementary Figure S50. a) MS (-*ve*) of the tetramer; zoom of the molecular ion is shown as an inset and b) MS/MS (-*ve*) of the tetramer. The species with *m/z* more than a dimer reinforces the idea of a macrocycle rather than a catenane.

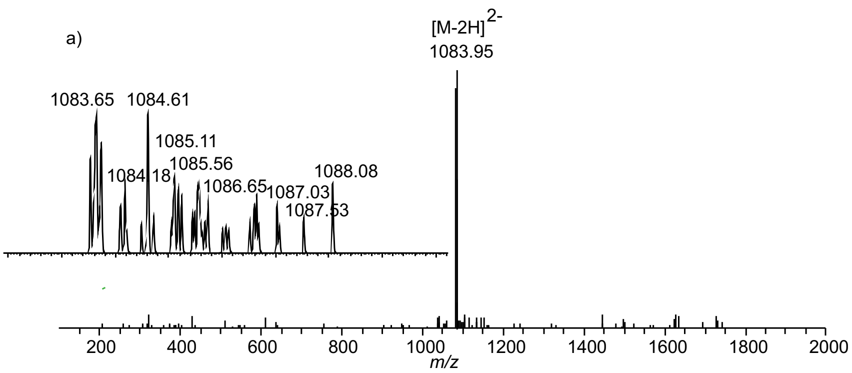

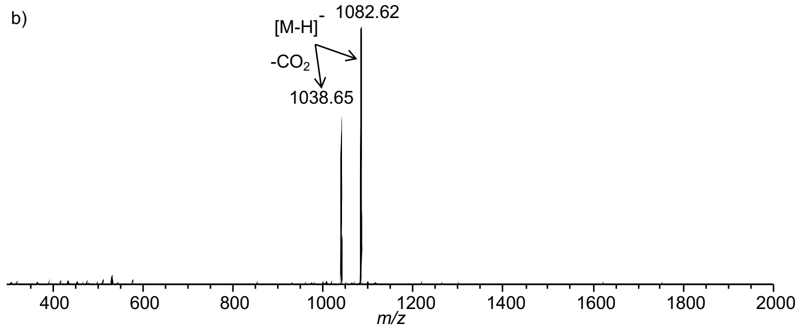

Supplementary Figure S51. a) MS (*-ve*) of the [2]catenane with mixed chiralities BDT; zoom of the molecular ion is shown as an inset and b) MS/MS (*-ve*) of the molecule.

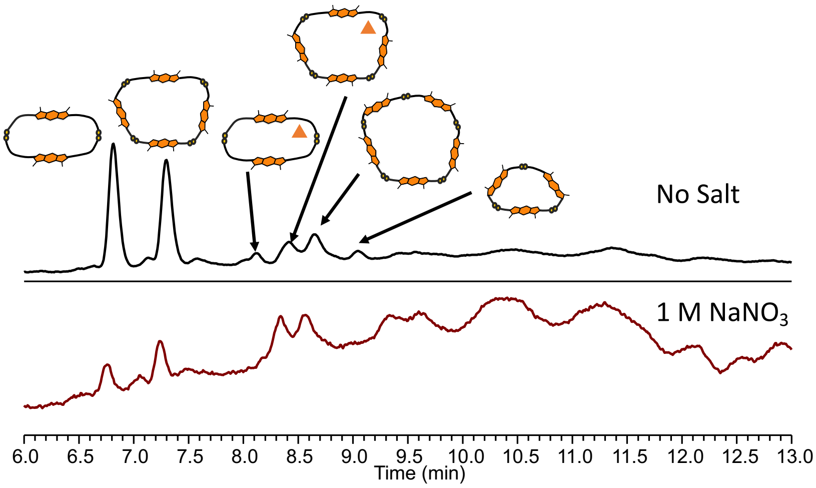

Supplementary Figure S52. Reverse-phase HPLC analysis of **2** (5 mM concentration) with a) no salt and b) in the presence of 1 M $NaNO_3$.

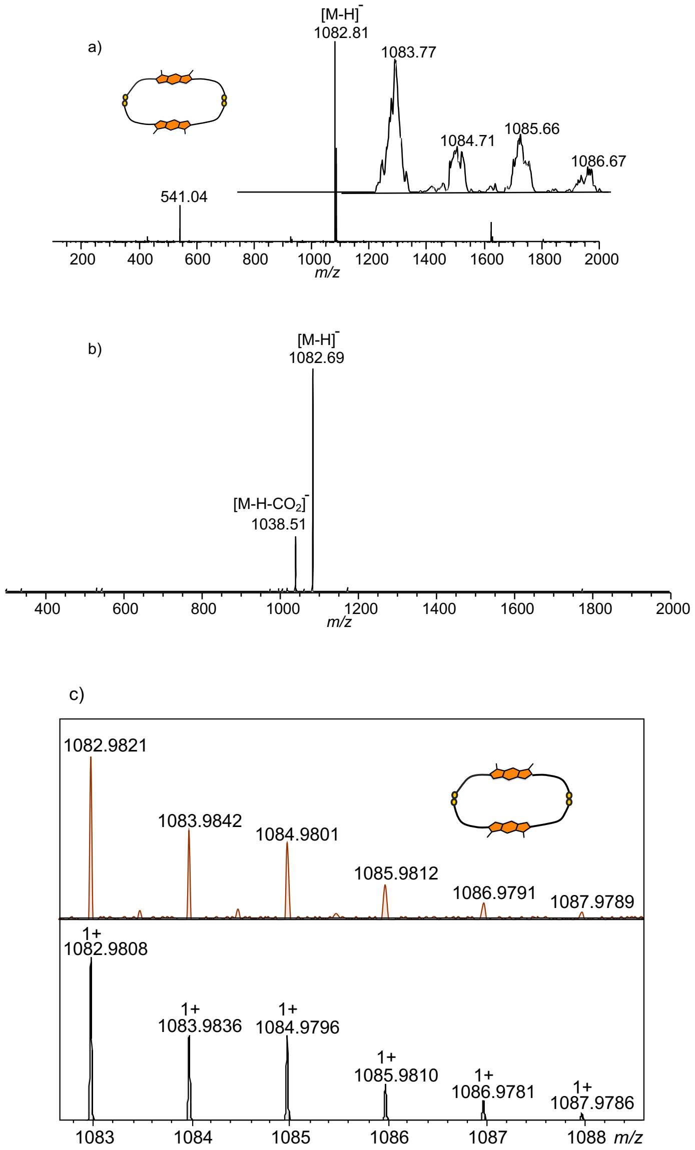

Supplementary Figure S53. a) MS (*-ve*) of **2 homodimer**; the zoom of the molecular ion is shown as an inset; b) MS/MS (*-ve*) of **2 homodimer** and c) HRMS of **2 homodimer** (top) and the simulated isotope pattern (bottom).

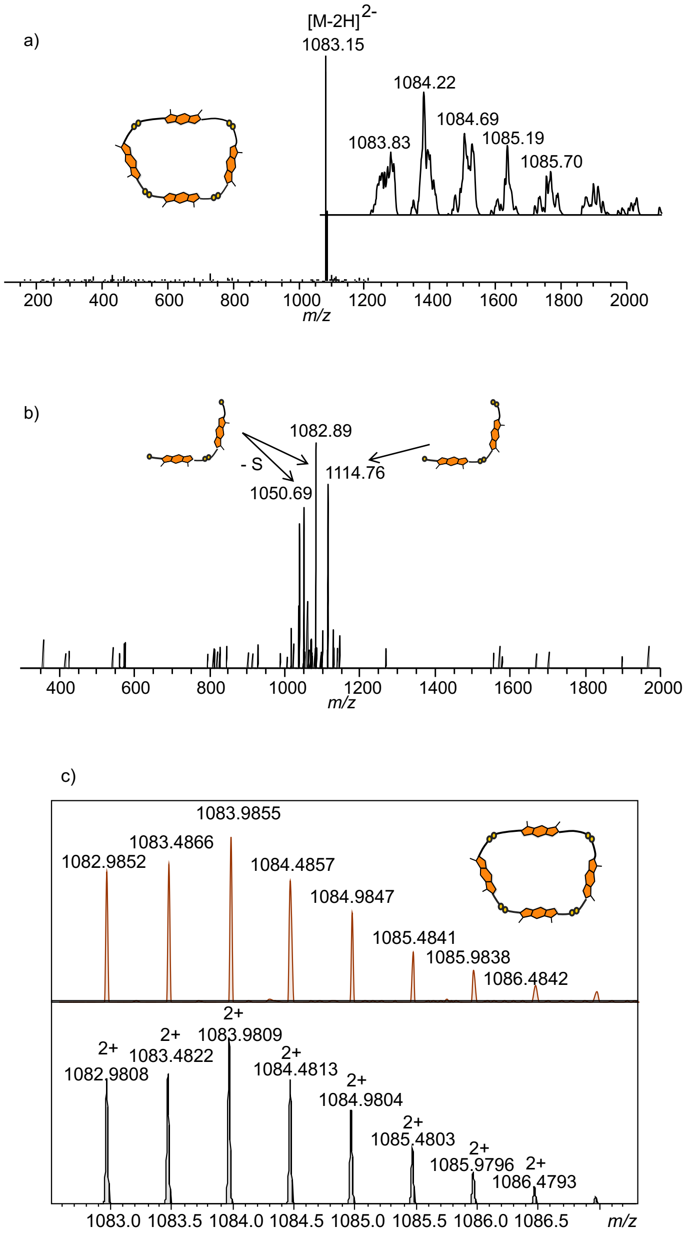

Supplementary Figure S54. a) MS (*-ve*) of **2 tetramer**; zoom of the molecular ion is shown as an inset; b) MS/MS (*-ve*) of **2 tetramer** and c) HRMS of **2 tetramer** (top) and the simulated isotope pattern (bottom).

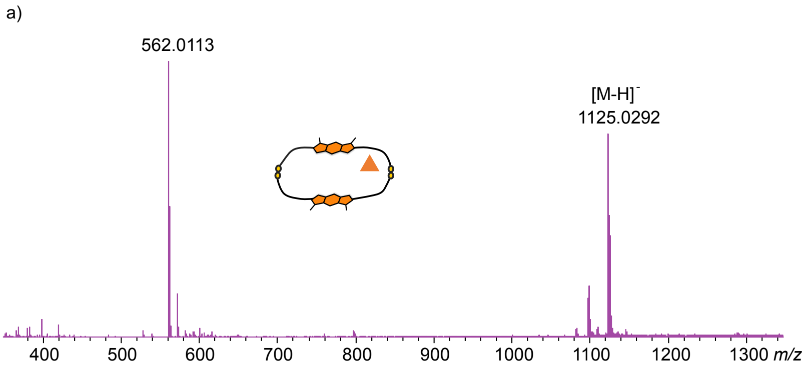

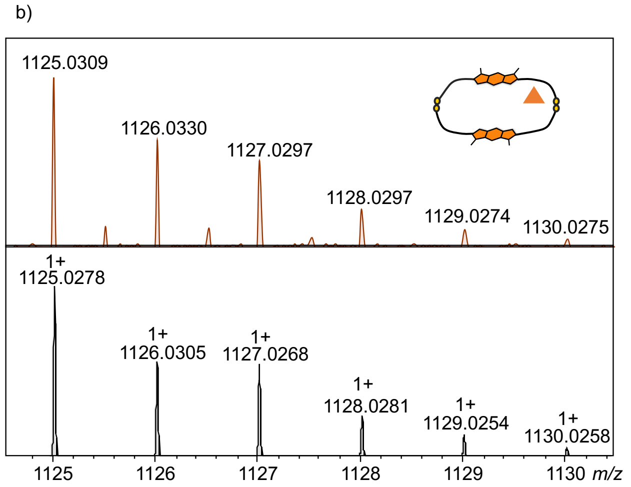

Supplementary Figure S55. a) MS (*-ve*) of **2 homodimer** with three additional methyl groups and b) HRMS of **2 homodimer** with three additional methyl groups (top) and the simulated isotope pattern (bottom).

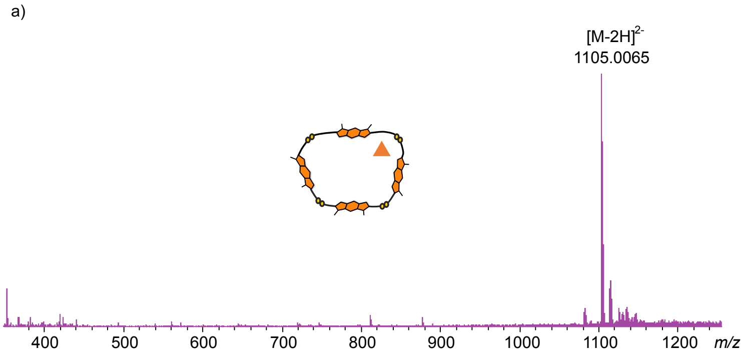

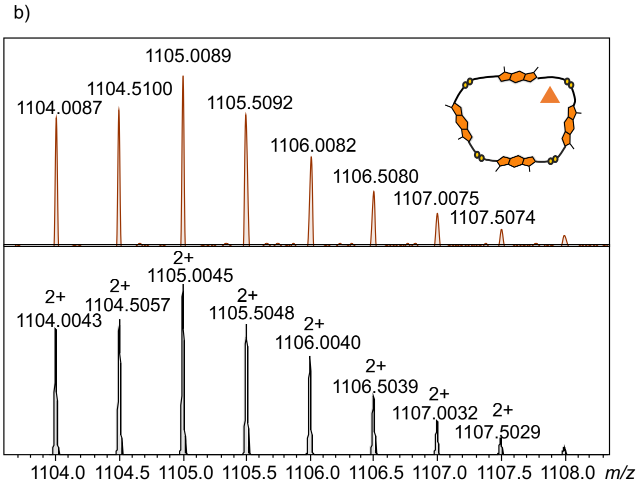

Supplementary Figure S56. a) MS (*-ve*) of **2 tetramer** with three additional methyl groups and b) HRMS of **2 tetramer** with three additional methyl groups (top) and the simulated isotope pattern (bottom).

a)

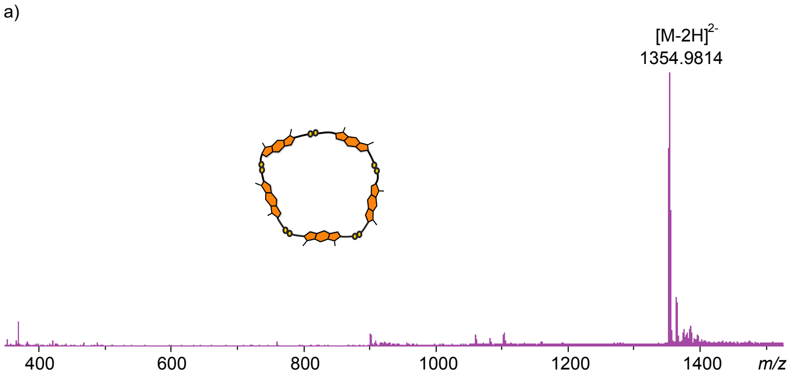

b)

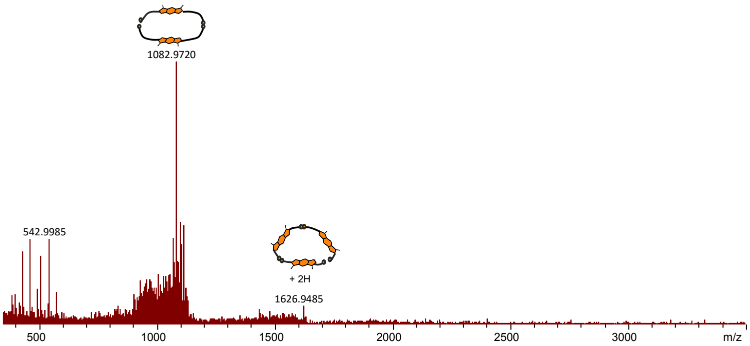

c)

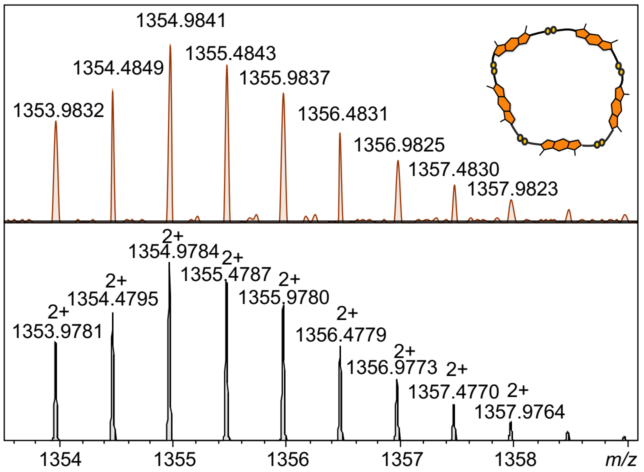

Supplementary Figure S57. a) MS (-*ve*) of **2 pentamer** and b) HRMS of **2 pentamer** (top) and the simulated isotope pattern (bottom) and c) MS/MS of **2 pentamer**.

a)

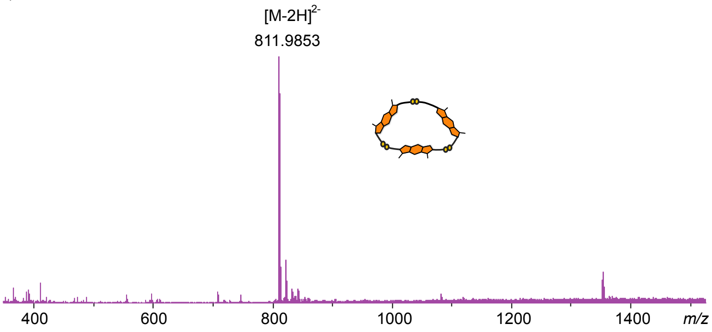

b)

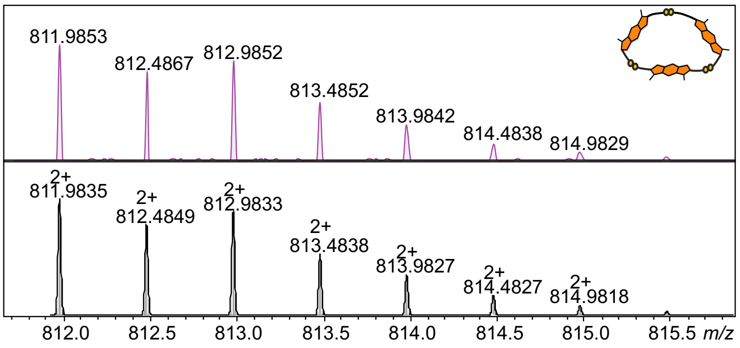

Supplementary Figure S58. a) MS (-*ve*) of **2 trimer** with three methyls and b) HRMS of **2 trimer** with three methyls (top) and the simulated isotope pattern (bottom).

Computational details:

Q_{zz} calculations were performed with GAMESS at MP2 level. Geometry optimisation was done with Avogadro[3] (Force field: UFF, Algorithm: Conjugate gradients. This was followed by MOPAC 2016[4] (version 18.117 M) PM7 semiempirical calculations using the COSMO water model and Gabedit 2.5.0[5] as interface.

The **1** and **2** homodimer molecular structures have been optimized using M06-2X/6-31G[6–8] as implemented in *Gaussian 16*,[9] using water as solvent *via* polarizable continuum model (PCM). Aromatic unit dimers (Figure S59) have been optimised in water using M06-2X/6-311G.[8,10]

The difference in enthalpies of formation of the 1 and 2 homodimers ($\Delta H_{BDT} - \Delta H_{iBDT} = -28.6\ kcal/mol$) have been computed (M06-2X/6-31G/water) by considering the energy of formation of the dimer and its constituent relaxed molecular units: $\Delta H = \sum H_{monomer} - H_{dimer} - 2H_{H_2}$.

ACID calculations have been performed for the M06-2X/6-311G[8,10] optimized geometries of BDT and iBDT molecular cores using the IOP(10/93=1) option implemented in *Gaussian 16*. Isosurface plots have been obtained using the ACID package.[11]

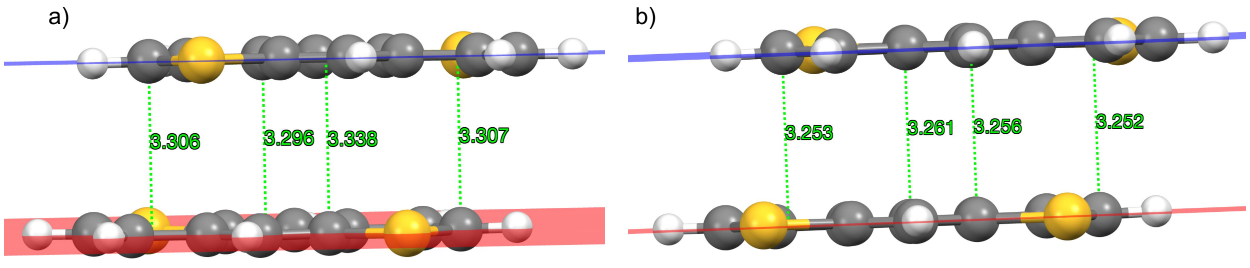

Supplementary Figure S59. Stacking distances between cores: a) BDT b) iBDT.

Molecular dynamics was done using Amber potential (in Gabedit 2.5.0), Conformational Search with the following parameters: Time (ps): Heating (1.5), Equilibrium (1), Production (10); Temperature (K): Heating (313), Production (313); Step size (fs): 1; Collapse (ps^{-1}): 20; Friction (ps^{-1}): 40; steps: NVE; MD Trajectory via Verlet velocity Algorithm with no constraints; Conjugate Gradients: Polak-Ribiere method. Each generated geometry was further optimized using PM7 and COSMO water parameters with gnorm of 0.01 (Tables S1 and S2).

Table S1: Energies of **1** homodimer conformations.

**1** homodimer protonated	Energy (kJ)	ΔE(E - E_{min})
1	-2656.82	1.01
2	-2636.79	21.04
3	-2628.45	29.38
4	-2638.2	19.63
5	-2634.69	23.14
6	-2657.83	0
7	-2631.04	26.79
8	-2637.86	19.97
9	-2630.9	26.93
10	-2637.69	20.14
11	-2629.86	27.97
12	-2651.79	6.04
13	-2655.51	2.32
14	-2639.42	18.41
15	-2634.43	23.4

Table S2: Energies of **2** homodimer conformations.

**2** homodimer protonated	Energy (kJ)	ΔE(E - E_{min})
1	-2634.84	1.3
2	-2631.19	4.95
3	-2632.68	3.46
4	-2632.4	3.74
5	-2632.14	4
6	-2629.95	6.19
7	-2632.74	3.4
8	-2630.98	5.16
9	-2631.2	4.94
10	-2622.21	13.93
11	-2619.83	16.31
12	-2621.04	15.1
13	-2620.21	15.93
14	-2636.14	0
15	-2618.03	18.11

Crystal data and structure refinement for 6 (CCDC: 2006236)

Empirical formula	$C_{58}H_{48}N_2O_8S_4 \cdot C_3H_5F_5O_2$	
Formula weight	1189.29	
Temperature	150.00(10) K	
Wavelength	1.54184 Å	
Crystal system	Orthorhombic	
Space group	$P2_12_12_1$	
Unit cell dimensions	a = 12.64620(10) Å	α = 90°.
	b = 17.88170(10) Å	β = 90°.
	c = 25.2987(2) Å	γ = 90°.
Volume	5720.94(7) $Å^3$	
Z	4	
Density (calculated)	1.381 Mg/m^3	
Absorption coefficient	2.172 mm^{-1}	
F(000)	2472	
Crystal size	0.180 x 0.180 x 0.100 mm^3	
Theta range for data collection	3.026 to 73.054°.	
Index ranges	-15<=h<=15, -19<=k<=22, -24<=l<=31	
Reflections collected	46206	
Independent reflections	11343 [R(int) = 0.0423]	
Completeness to theta = 67.684°	99.8 %	
Absorption correction	Semi-empirical from equivalents	
Max. and min. transmission	1.00000 and 0.95696	
Refinement method	Full-matrix least-squares on F^2	
Data / restraints / parameters	11343 / 40 / 832	
Goodness-of-fit on F^2	1.034	
Final R indices [I>2sigma(I)]	R_1 = 0.0378, wR_2 = 0.0962	
R indices (all data)	R_1 = 0.0404, wR_2 = 0.0984	
Absolute structure parameter	0.007(5)	
Largest diff. peak and hole	0.486 and -0.345 $e \cdot Å^{-3}$	

References:

1. Ota, S. *et al.* Synthesis and properties of a benzo[1,2-b:4,5-b′]dithiophene core π-system that bears alkyl, alkylthio and alkoxy groups at 3,7-positions. *RSC Adv.* **3**, 12356–12365 (2013).
2. Shi, Y. *et al.* Highly Active Catalysts for the Transfer Dehydrogenation of Alkanes: Synthesis and Application of Novel 7-6-7 Ring-Based Pincer Iridium Complexes. *Chem. - Eur. J.* **19**, 10672–10689 (2013).
3. Hanwell, M. D. *et al.* Avogadro: an advanced semantic chemical editor, visualization, and analysis platform. *J. Cheminformatics* **4**, 17 (2012).
4. Stewart, J. J. P. Optimization of parameters for semiempirical methods VI: more modifications to the NDDO approximations and re-optimization of parameters. *J. Mol. Model.* **19**, 1–32 (2013).
5. Allouche, A.-R. Gabedit-A graphical user interface for computational chemistry softwares. *J. Comput. Chem.* **32**, 174–182 (2011).
6. Becke, A. D. Density-functional thermochemistry. III. The role of exact exchange. *J. Chem. Phys.* **98**, 5648–5652 (1993).
7. Lee, C., Yang, W. & Parr, R. G. Development of the Colle-Salvetti correlation-energy formula into a functional of the electron density. *Phys. Rev. B* **37**, 785–789 (1988).
8. Hehre, W. J., Ditchfield, R. & Pople, J. A. Self—Consistent Molecular Orbital Methods. XII. Further Extensions of Gaussian—Type Basis Sets for Use in Molecular Orbital Studies of Organic Molecules. *J. Chem. Phys.* **56**, 2257–2261 (1972).
9. Gaussian 16, Revision A.03, M. J. Frisch *et al.* , Inc., Wallingford CT, 2016.
10. Zhao, Y. & Truhlar, D. G. The M06 suite of density functionals for main group thermochemistry, thermochemical kinetics, noncovalent interactions, excited states, and transition elements: two new functionals and systematic testing of four M06-class functionals and 12 other functionals. *Theor. Chem. Acc.* **120**, 215–241 (2008).
11. Herges, R. & Geuenich, D. Delocalization of Electrons in Molecules †. *J. Phys. Chem. A* **105**, 3214–3220 (2001).
